# Lidar survey of ancient Maya settlement in the Puuc region of Yucatan, Mexico

**DOI:** 10.1371/journal.pone.0249314

**Published:** 2021-04-28

**Authors:** William M. Ringle, Tomás Gallareta Negrón, Rossana May Ciau, Kenneth E. Seligson, Juan C. Fernandez-Diaz, David Ortegón Zapata

**Affiliations:** 1 Dept. of Anthropology (Emeritus), Davidson College, Davidson, North Carolina, United States of America; 2 Centro Regional Yucatán, Instituto Nacional de Antropología e Historia, Mérida, Yucatán, México; 3 Bolonchen Regional Archaeological Project, Oxkutzcab, Yucatán, México; 4 Department of Anthropology, California State University Dominguez Hills, Carson, California, United States of America; 5 National Center for Airborne Laser Mapping, University of Houston, Houston, Texas, United States of America; Universita degli Studi di Milano, ITALY

## Abstract

The application of lidar remote-sensing technology has revolutionized the practice of settlement and landscape archaeology, perhaps nowhere more so than in the Maya lowlands. This contribution presents a substantial lidar dataset from the Puuc region of Yucatan, Mexico, a cultural subregion of the ancient Maya and a distinct physiographic zone within the Yucatan peninsula. Despite the high density of known sites, no large site has been fully surveyed, and little is known about intersite demography. Lidar technology allows determination of settlement distribution for the first time, showing that population was elevated but nucleated, although without any evidence of defensive features. Population estimates suggest a region among the most densely settled within the Maya lowlands, though hinterland levels are modest. Lacking natural bodies of surface water, the ancient Puuc inhabitants relied upon various storage technologies, primarily *chultuns* (cisterns) and *aguadas* (natural or modified reservoirs for potable water). Both are visible in the lidar imagery, allowing calculation of *aguada* capacities by means of GIS software. The imagery also demonstrates an intensive and widespread stone working industry. Ovens visible in the imagery were probably used for the production of lime, used for construction purposes and perhaps also as a softening agent for maize. Quarries can also be discerned, including in some cases substantial portions of entire hills. With respect to agriculture, terrain classification permits identification of patches of prime cultivable land and calculation of their extents. Lidar imagery also provides the first unequivocal evidence for terracing in the Puuc, indeed in all northern Yucatan. Finally, several types of civic architecture and architectural complexes are visible, including four large acropolises probably dating to the Middle Formative period (700–450 B.C.). Later instances of civic architecture include numerous Early Puuc Civic Complexes, suggesting a common form of civic organization at the beginning of the Late Classic demographic surge, (A.D. 600–750).

## Introduction

**Fig 1:**
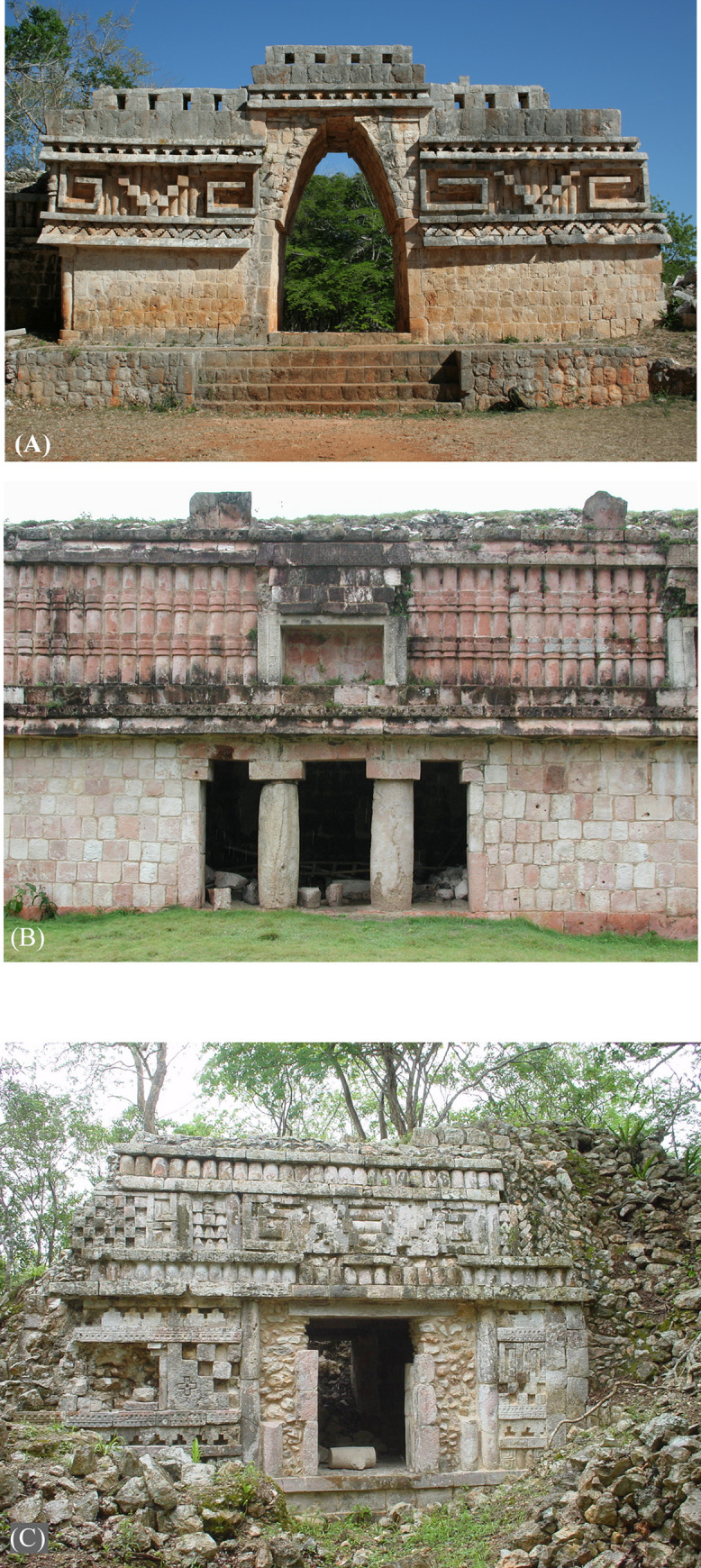
Typical examples of Puuc masonry architecture. (A) Labna. (B) Chacmultun. (C) Rancho Pérez.

**Fig 2:**
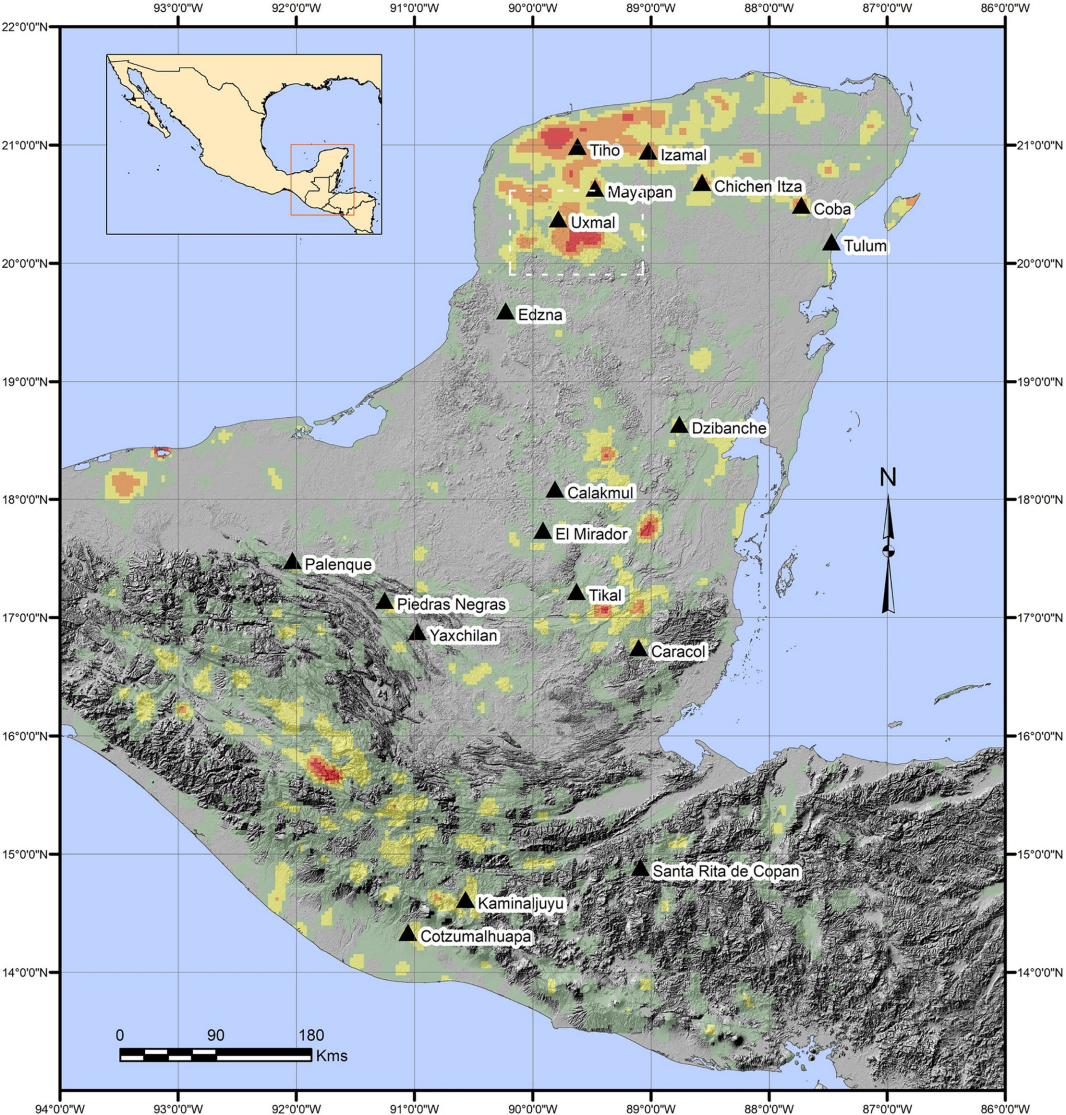
Site density in the Maya area. Red signifies zones of high density. Because survey coverage is uneven, and no attempt is made to account for site size or date of occupation, this should not be taken as a representation of actual population density. (Based upon the *Electronic Atlas of Ancient Maya Sites* [8]; data courtesy of Walter Witschey).

Since the pioneering explorations of John Lloyd Stephens and Frederick Catherwood in the 1840s [1], the Puuc has been recognized as a regional variant of ancient Maya civilization, primarily on the basis of a common architectural style (Fig 1). Hundreds of sites are now known from archaeological and architectural surveys over the succeeding 175 years [2–7], marking this as one of the most densely settled regions of the Maya lowlands in terms of known sites, although most are relatively modest in size, rarely more than a few square kilometers in extent (Fig 2). The overwhelming majority (well over 90%) of visible Puuc archaeological features date to the Late-Terminal Classic periods (c. AD 600–950), although communities over a millennium older are known. Despite this long history of scholarship, fine-grained settlement studies are rare, while assessments of intersite population densities are virtually unknown.

**Fig 3:**
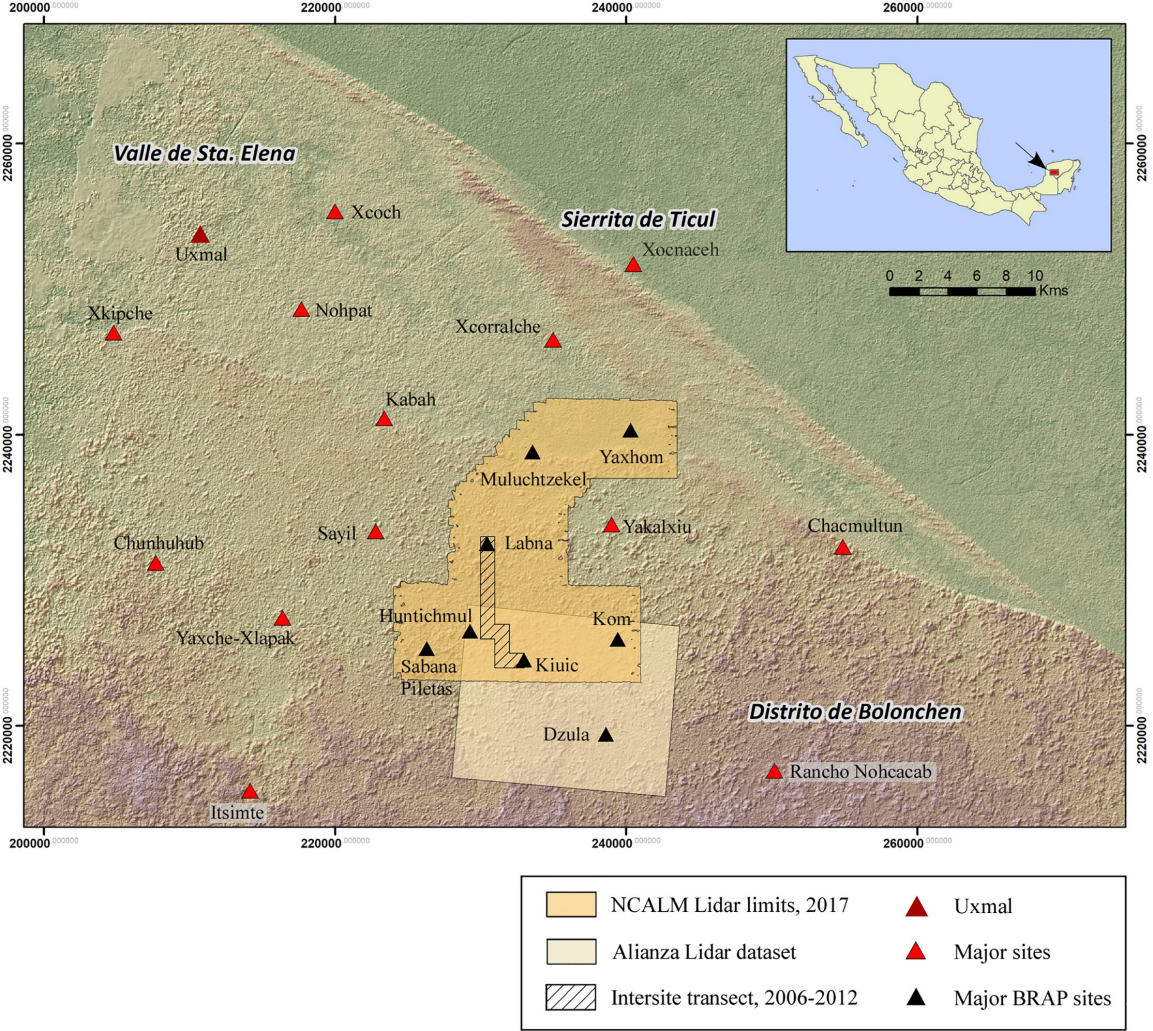
Map of the Puuc region, Yucatan, Mexico.

The Puuc is also a distinct physiographic zone of the Yucatan peninsula, whose northern limit is marked by a low escarpment known as the Sierrita de Ticul, a ridge of hills rarely more than 110–120 m above the plains to the north (Fig 3). To the south of the escarpment is an uplifted wedge of rolling terrain (Duch Gary’s [9] *lomerío bajo con llanuras*). Often the swales between higher ground are filled with soil, resulting in the formation of “flats” of relatively deep soils that are today favored for agriculture (referred to as *planadas* herein). This zone widens from east to west and is known as the Valle de Sta. Elena, after the largest town within it. Within the Valle de Sta. Elena (VSE) is a shallow basin known locally as the Valle de Yaxhom, important to the study that follows. Finally, a dense zone of kegelkarst or conekarst hills (Duch Gary’s *lomerío alto con llanuras*), known as the Distrito de Bolonchen (DBC), lies to the south of the VSE. Thousands of such hills divide the landscape, although they hardly ever rise more than 90 m above the surrounding terrain. Bordering the DBC to the south is a zone of seasonal swamps (*ak’alches*) whose archaeology is understudied [10]. It is important to note that there are basins of varying sizes and uplifted areas bordering them in each of these broad zones, and that soil cover in uplifted areas is usually relatively shallow, enhancing the visibility of archaeological features.

This paper is an initial report on the results of a lidar (airborne light detection and ranging) mission flown over a portion of the Puuc in 2017, the first such sample from the region collected specifically for archaeological purposes and one of the few that have been flown over northern Yucatan [11–15]. These data were collected in support of the Proyecto Arqueológico Regional de Bolonchén (PARB), co-directed by Gallareta Negrón, Ringle, and Dr. George Bey of Millsaps College. Since 2000 we have been examining Puuc settlement from a variety of perspectives and at a number of sites, though the addition of lidar data has vastly enlarged our frame of reference, from less than 15 km^2^ to over twenty times that. An overarching question we hoped to address by means of lidar data is whether the regionalism evident in architecture could also be detected in its settlement patterns. Could settlement differences be attributed, at least in part, to the distinctive landscape in which they arose, and at a finer level, could differences be detected between the two major physiographic zones of the Puuc, the VSE and DBC, given the impediments to settlement within the hill district? Was the extreme site density mentioned above also true at the household level, or were sites mainly small clusters of elite buildings, such as has been observed in the Rio Bec subregion [16], an area sometimes considered to be ancestral to the Puuc. Clearly the Puuc style spread to other parts of Yucatan, such as Chichen Itza, Ek Balam, Kuluba, and elsewhere, but might the remarkably homogenous Puuc architecture reflect a common form of civic organization?

We also address a number of sub-themes. What was the carrying capacity of the area, given what we know of contemporary farming practices, and how might land have been managed? What do settlement data indicate regarding population levels? What use was made of the hills, both as a resource and locus of settlement? How did people move between and communicate across this complex karst landscape? The availability of a large, statistically significant sample of the Puuc landscape allows such themes to be addressed quantitatively for the first time by means of GIS analyses of digital lidar elevation imagery.

## Methods: The lidar dataset

Lidar data collection and fieldwork were carried out under the auspices of the Proyecto Arqueológico Regional de Bolonchén. Permission to work was obtained from the Consejo de Arqueología of the Instituto Nacional de Antropología e Historia (Oficio 401.1S.3-2017/650 for the lidar collection and Oficios 401.1S.-2017/1387, 401.1S.3-2018/1256, and 401.1S.3-2019/1222 for fieldwork in 2017, 2018, and 2019 respectively). The lidar dataset was collected by the National Center for Airborne Lidar Mapping (NCALM) on May 5 and 7, 2017, employing the Teledyne Optech Titan multispectral lidar sensor [17]. The sensor was flown on a Piper PA-31 twin engine aircraft at an altitude of 600 m above ground level and an average ground speed of 70 m/s. The sensor was configured with a total pulse repetition frequency of 450 kHz (150 kHz per channel) and the scanner running at 25 Hz with a ± 28° scan angle. Early May is usually one of the hottest and driest times of the year in Yucatan, and 2017 was no exception. Today the Puuc is largely covered by medium-height deciduous tropical forest (*selva mediana caducifolia* [18]), hence most of the foliage had fallen and visibility was generally excellent. The flight resulted in a total of 237.23 km^2^ of usable data, although coverage of the fringes beyond the requested 185 km^2^ does contain several minor gaps and is not at the nominal lidar densities carefully controlled within the main survey area. Although point cloud accuracy was not directly assessed for the project area, it is estimated than the vertical precision of the returns is better than 8 cm and the horizontal precision better than 20 cm. Other general technical specifics of our dataset are provided in Table 1.

**Table 1:** Lidar data collection parameters*.

Collection Dates:	Two Flights flown on May 04 and 05, 2017 (DOY 124 and 125), Flight 1: Lines 1–47, Flight 2: Lines 48–64
Sensor:	Teledyne Optech Titan MW (14SEN/CON340)
Flight parameters:	Flying height: 600 m AGL, Swath width: 300 m, Overlap: 50%, Line spacing: 600 m
Equipment parameters:	PRF: 150 kHz per channel (450 kHz total), Scan Frequency: 25 Hz, Scan Angle: ± 28°, 2° cutoff in processing.
Nominal Laser Pulse (Shot) Density:	21 pulses/m^2^.
Requested/Collected Area:	185.0/237.2 km^2^
Reference Station Summary:	20.2958356722 N, 89.41093833888 W, 24.438 m (Ellipsoid), used for flights 1 and 2
Horizontal and Vertical Datum:	IGS08 (EPOCH:2010.0000) / WGS84 Ellipsoid Heights
LAS format:	1.4
DTM/DEM resolution	0.5 m

* Data provided by NCALM as part of the project metadata report.

NCALM supplied the data in the form of DTM and DSM rasters with a resolution of .5 m, as well as the classified point cloud dataset. (DTM refers to a digital terrain model, often referred to as a “bare earth” raster. A DSM is a digital surface model usually created from lidar first returns and hence reflects the tops of the tallest features, such as treetops or the tops of crops or scrub vegetation but bare earth where there is no ground cover. We employ DEM, digital elevation model, to refer to any raster elevation surface.) We found that the supplied point classification, done with TerraScan software, yielded significantly more ground points than several others we created using alternative software, among them ArcGIS 10.5 and LASTools. Experiments with regenerating rasters at a finer resolution showed no measurable improvement. A “problem” common to all these packages is that the points corresponding to some ancient standing buildings, like their modern equivalents, were classified as “6-building” and so were not part of the DTM. These, however, could be manually reclassified and inserted as patches to the master DTM, although because they almost always rest on a basal platform, this did not affect their inclusion in the feature count.

The generally favorable conditions for lidar collection in the Puuc are supported by point cloud classification statistics (Table 2). By way of comparison, about 62.3% of our pulses produced ground returns, and 27.1% of our returns were classified as ground returns, versus 14.2% of the pulses and 10.3% of the points for the initial data gathered over Chichen Itza and Yaxuna in 2014 [14]. Values for the 2016 Pacunam campaign over northern Guatemala are 15.4% and 8.7% respectively [19]. Puuc ground return density was about 10.6/m^2^, versus 2.4/m^2^ for the Guatemalan campaign and about 2 for the 2014 Chichen Itza-Yaxuna dataset (this last is not stated directly; the value given is the number of ground returns divided by the area covered). Stanton et al. [15] do not report pulse statistics for the most recent Coba-Chichen Itza dataset, but because it was collected with the same sensor as our project, their values are presumably comparable. Ground return density at Mayapan [10] was higher, 11.4/ m^2^, but it was also collected at a much higher pulse density (> 40 pulses/m^2^) specifically to enhance imagery definition.

**Table 2:** Lidar point cloud statistics.

Classification	Returns (Points)	Return %	Return Density/m^2^*
unclassified (1)	12,754,856	0.14%	0.1
ground (2)	2,506,150,080	27.10%	10.6
low vegetation (3)	372,649,902	4.03%	1.6
medium vegetation (4)	2,317,106,211	25.05%	9.8
high vegetation (5)	4,040,215,215	43.68%	17.0
building (6)	112,672	0.00%	0.0
noise (7)	23,982	0.00%	0.0
**Totals**	**9,249,012,918**	**100.00%**	**39.0**
**Total laser pulses**	**4,017,097,894**		

*Total lidar coverage: 237.2 km^2^.

Two other lidar datasets, both collected for forestry and ecology purposes, partially overlap our study area. In 2013, as part of its AMIGA-Carbproject, NASA use its G-LiHT sensor suite to fly three narrow transects that partially impinged on our study area [20–22]. About 21 km^2^ extend beyond the limits of the NCALM dataset. A second dataset, which we only became aware of while completing this manuscript, dates from the following year and was collected by the Alianza MexicoREDD+, a group of organizations carrying out forestry and carbon storage research in Mexico (ereddplus.com). This dataset totals 174.2 km^2^, 116.8 km^2^ of which extends beyond the bounds of our dataset to the south and southeast and completely subsumes the G-LiHT transects. Thus, we have a total of 354.1 km^2^ of lidar coverage, although since we have only begun examination of the Alianza dataset, this discussion will deal almost exclusively with our original NCALM data. Any reference to the Alianza dataset concerns only the 116.8 km^2^ exterior to the NCALM boundaries.

Although the horizontal alignment of the three datasets is excellent, their elevations differ non-uniformly because they were processed with different references (geoids & ellipsoids) and because of the vagaries of the point clouds (i.e., it is not simply a matter of applying a uniform elevation offset to a given dataset). The NASA and Alianza DTMs also had to be reclassified and resampled, since they were imaged at 1 m resolution, and visual inspection indicated that several peaks had been truncated during classification. Reclassification was done by the senior author using the ArcGIS 10.5 tool “Classify LAS Ground” in two passes, using the Conservative and Aggressive settings, the latter with the “Reuse Existing Ground” option. Although we are just beginning analysis, it is evident that both the Alianza and NASA datasets have considerably more noise than the NCALM data, probably due to the newer sensor utilized by NCALM. NCALM has also started the reprocessing of these datasets using their procedures and software to better match our 2017 dataset.

**Fig 4:**
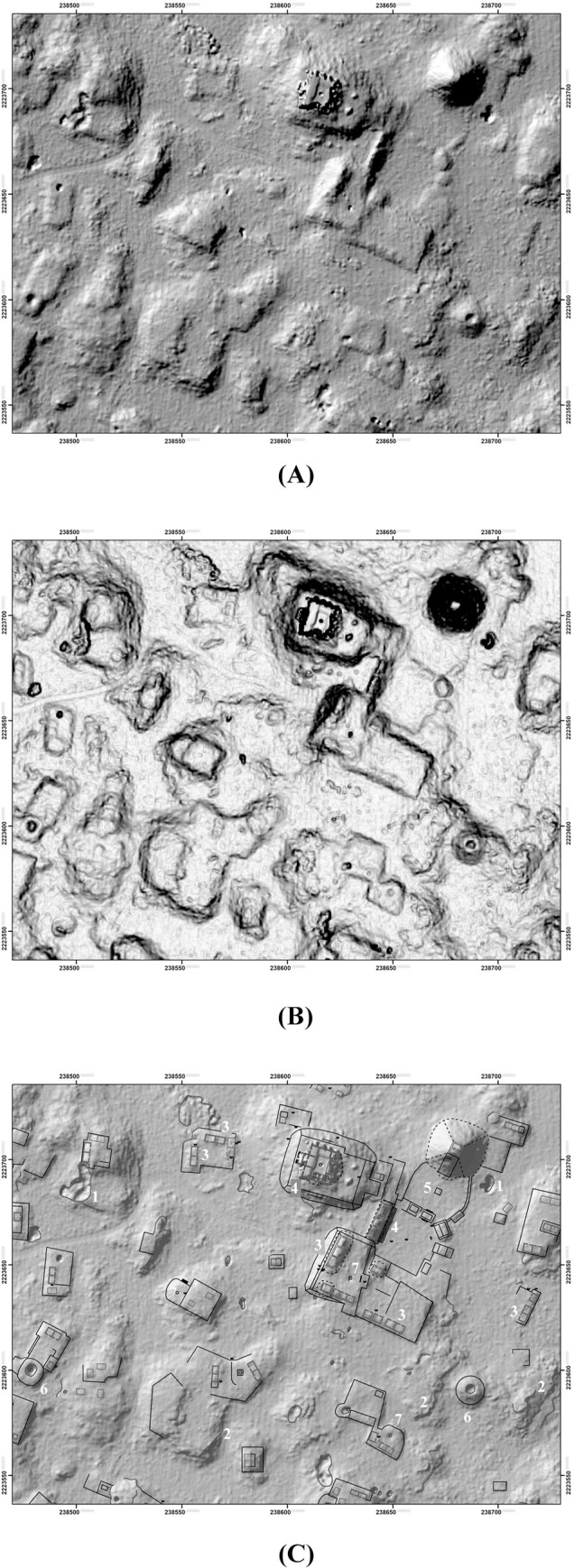
Methods of data visualization. (A) Hillshade. (B) “Slope-shade” filters. (C) CAD overlay of structures identified by terrestrial survey. 1 = Pit quarry, 2 = ledge quarry, 3 = perishable frame brace, 4 = masonry building, 5 = EPCC, 6 = annular structure, 7 = chultun/chultun platform. Only a few representatives are labeled. Note the difficulty of distinguishing frame braces in the lidar images.

Features were identified by the senior author, who has considerable field experience in Puuc settlement archaeology. The imagery was examined at a scale of approximately 1:1000, less if necessary, and features were registered by a central point. The data were completely rechecked in preparation for this article. Standard hillshade images (315° Azimuth, 45° Elevation) were our primary method for visualizing the data. These were paired with “slope shade” surfaces based upon slope and colored with a gray-scale color ramp. Whereas the unidirectional illumination of standard hillshades can sometimes obscure features, the slope filter essentially highlights the sloping outline of structures with dark coloration, while conversely flat surfaces, such as the tops of platforms, are lighter (Fig 4). In doubtful cases, toggling between the two helped us decide whether to include features, which were then recorded by means of a point shapefile.

Architectural terminology can vary within Maya archaeology and is sometimes loosely applied. For consistency, “structure,” when unmodified, is used herein as the general term encompassing platforms, buildings (defined as a roofed area), and annular structures. The features used in our analysis are those platforms and buildings resting directly upon the ground (annular structures, described later in this article, were tallied separately). The great majority of these features are basal platforms built of stone rubble, which may in turn support additional buildings, secondary platforms, or other types of construction (which are not tallied). Basal platforms range in size from a few to over a hundred meters on a side, and given the thinness of soil cover, are usually readily visible in the imagery.

In some cases, buildings rest directly on the ground, without a basal platform, and thus potentially form part of the tally. Buildings with walls and roofs of masonry construction, especially if they had stone vaulted roofs, are relatively easy to identify in the imagery, since they collapse into mounds 1–4 m high and 5–20+ m in length. (Though buildings on platforms are not included in the feature tally, we have found the open negative filter of the Sky-View Factor Lidar toolkit [23, 24] to be useful to identifying these mounds, while the open positive filter highlights standing walls and rooms within partially collapsed masonry buildings.) In contrast, those buildings primarily of pole and thatch construction survive only as foundation walls at most a few courses high. These cannot be consistently distinguished in the imagery (Fig 4C), hence our choice of basal features as the unit of analysis. Admittedly, some small buildings resting on the ground cannot be identified in the lidar and are thus missed in our tally, but the percentage of such features is much less than if we had made buildings, on or off platforms, our fundamental unit of analysis. For similar reasons, the Caracol project in central Belize also used basal structures, again mostly platforms, as the fundamental unit in studies of its lidar imagery [25], as have most of the projects in northern Yucatan [13, 14].

**Fig 5:**
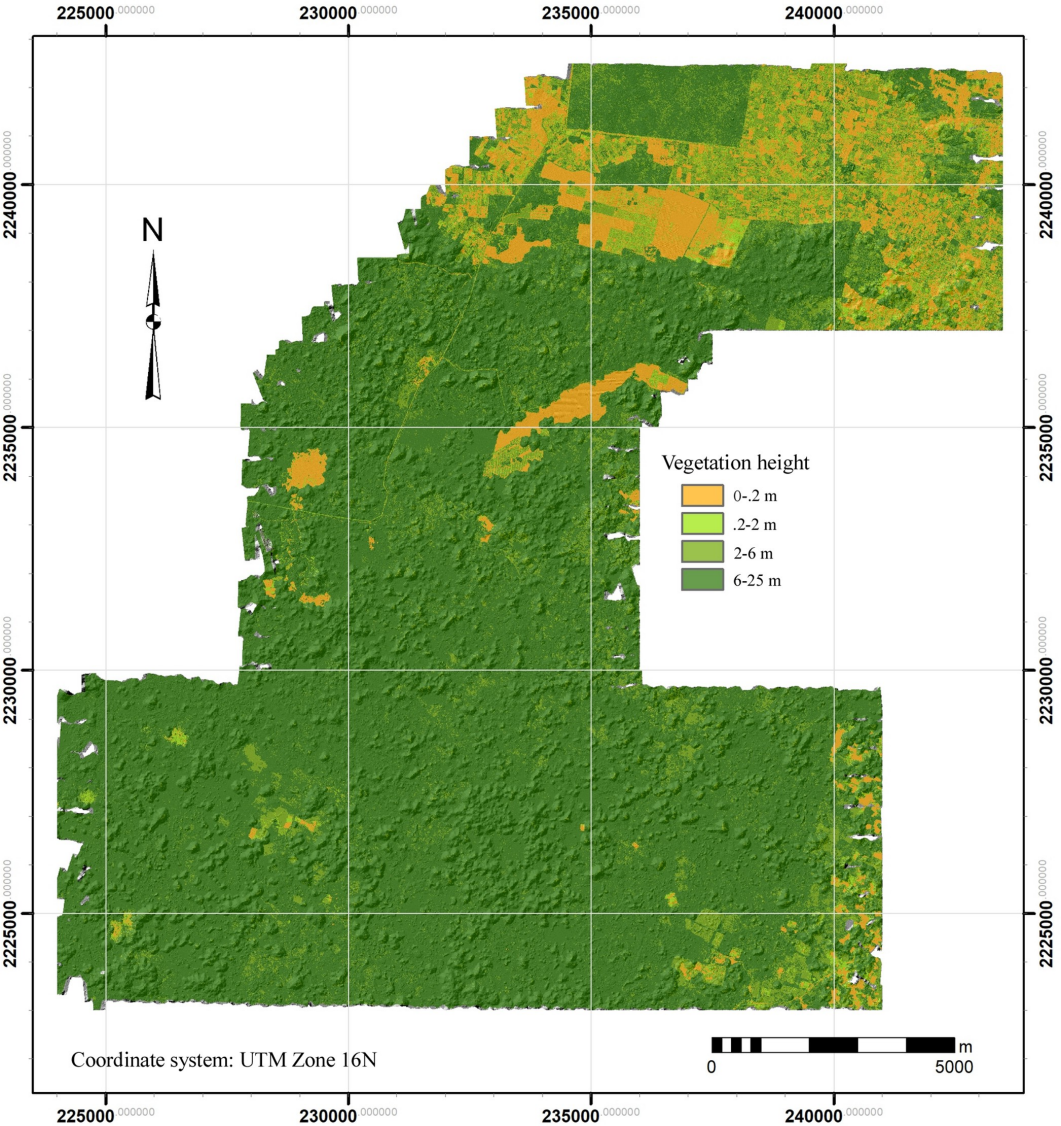
Vegetation classified by height in the NCALM sample (difference of the DSM and DTM raster layers). The greater part is medium tropical deciduous forest, in dark green.

Earlier lidar projects in northern Yucatan experienced some difficulty recognizing features where scrub vegetation predominates [12, 14]. As Fernandez-Diaz et al. [26] states, this occurs when vegetation height is less than the lidar range resolution. Stanton et al. [15] noted a marked improvement in a second lidar dataset collected with the newer Titan MW sensor at the height of the dry season, due in part to its shorter range resolution. Our dataset was collected during the same campaign, and we too have had relatively few problems with lower growth, although this has as much to do with the nature of the ground cover. As noted, deciduous tropical forest predominates, but today fields are increasingly being cleared for commercial agriculture. A calculation of ground cover height, made by subtracting our DTM raster from the DSM raster and then smoothing to a grid of 5m, reveals about 5.5% of the area is essentially bare earth, 5.1% is vegetation 2 m or less in height, and a further 17.5% is between 2 to 6 m (Fig 5, Table 3). The remainder, nearly 72%, is forest. The fact that point cloud statistics indicate that only 4.03% of returns were classified as low vegetation (< 3m) while 68.7% was either medium or high vegetation (> 3m), suggests that interference from low vegetation was relatively minor below the tree canopy. The problems we encountered occur mainly in areas recently returned to fallow (*hubche*) or covered in low crops, such as orchards of fruit trees whose foliage extended almost to the ground.

**Table 3:** Current land cover of the survey area.

Vegetation Height*	5 m Raster Cells	Area (Km^2^)	% of Total Land Cover
0-.1	510,460	12.762	5.50%
.1–2	472,703	11.818	5.09%
2–6	1,622,921	40.573	17.49%
6–10	4,116,851	102.921	44.37%
10–15	2,464,137	61.603	26.56%
15–20	89,979	2.249	0.97%
above 20	1,096	0.027	0.01%
	9,278,147	231.954	

* Calculated by subtracting the regional DTM (bare earth) raster from the DSM (canopy surface) and then generalizing to 5 m cells.

### Puuc settlement research

In addition to the studies mentioned earlier, previous settlement studies in the Puuc include early work by Edward Thompson at Labna in the late 1880s [27], the first attempt to identify commoner residences, and a survey by the Carnegie Institution in the 1930s and 1940s focused on standing architecture but with some mapping of the major site centers [3]. The first modern settlement study, also site-centered, was not carried out until the 1980s. This was at Sayil [28, 29], a Rank 2 center. Several of the project alumni then went on to work at other sites within the region, such as Smyth’s work at Chac II [30] and more recently Xcoch [31, 32]. Other site-focused settlement studies include projects at Xculoc and Xcalumkin [33, 34], at Xkipche [35, 36], and at Labna [37, 38]. Work by Stephan Merk [39] and Antonio Benavides et al. [40] touches on the southwest extreme of our survey sample but mostly concerns civic architecture. Government-sponsored megaprojects have been ongoing at Uxmal and Kabah for many years, but only limited settlement information from Kabah and Uxmal are as yet available [41, 42]. Prior to our work, the only systematic assessment of intersite settlement was a transect between Xculoc and Chunhuhub carried out in 1987–1988, totaling about 5.3 km in length and about 100 m in width, although it narrowed to 10–20 m over the final 2.38 leg to Chunhuhub [34]. Relatively little was encountered within this transect.

Systematic site inventories of the Puuc began in the 1970s, when the *Atlas Arqueológo de Yucatán* [5] compiled the first reasonably complete compendium of sites within the state of Yucatan, 151 of which were within the Puuc. Nicholas Dunning [4] registered a total of 230 sites, some added as the result of his own fieldwork and others by Kurjack and Gallareta Negrón. His study stands out for its insights into the regional ecosystem, especially soils, although given the resources at his disposal he was only able to partially survey most sites. The most recent estimate, by the Maya Atlas project [8], has just under 400 sites registered in the region, including those in Campeche not included in the *Atlas Arqueológico*.

We began the Bolonchen Regional Archaeological Project (PARP) in 2000, initially focused on the site of Kiuic but soon with subprojects at a number of other nearby sites. Bey and colleagues began long-term intensive excavations of an Early Puuc civic/residential complex, the Yaxche Group, in that year and subsequently extended excavations to include an outlying hilltop elite complex called the Escalera al Cielo [43]. Most recently, his group has started work on a later civic complex at Kiuic, the Grupo Kuche. Ringle mapped Kiuic from 2000–2003 and then moved survey to Huntichmul, a larger center about 4.5 km NE of Kiuic. In 2000, Gallareta Negrón and Ramon Carrillo Sánchez began a trail survey between Kiuic and Labna, about 8.5 km NNE of Kiuic, to assess intersite settlement. In 2006, Gallareta and May Ciau redesigned this as a 1-km-wide, 11.5-km-long transect connecting Kiuic, Huntichmul, and Labna, which they have surveyed in its entirety. Concluded in 2012, this provided by far the largest sample of intersite settlement from the DBC as of 2017 [44]. To provide comparable information from the *lomerío bajo*, beginning in 2010 Ringle directed survey and limited excavations in the Valle de Yaxhom, assisted by Seligson, Melissa Galván Bernal, Gabriel Tun Ayora, and Ortegón Zapata.

**Fig 6:**
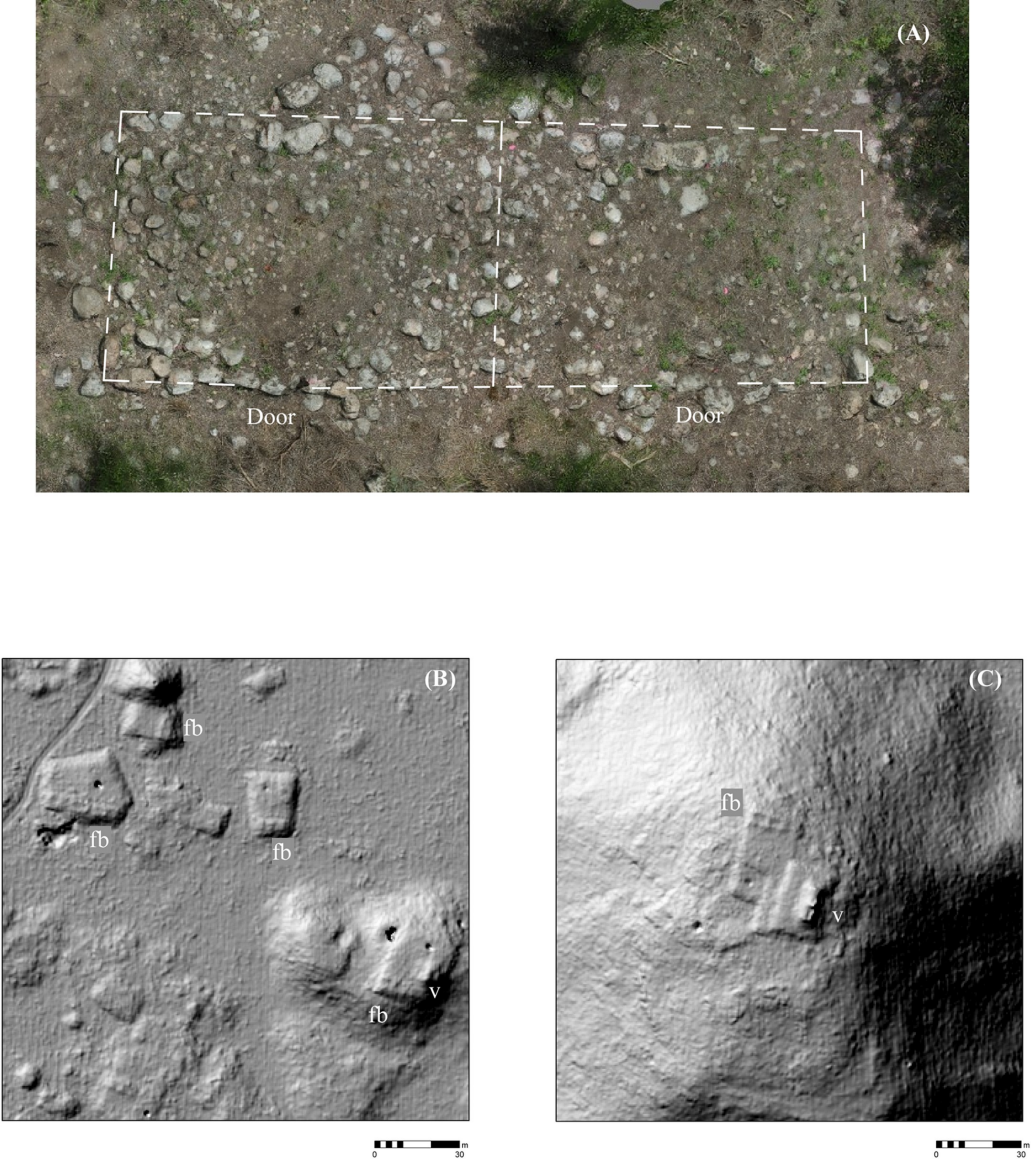
Frame braces of perishable housing. (A) An archaeological example cleared for mapping (not visible in the lidar). (B) examples from Acambalam II/III. (C) a particularly well-defined example from Kiuic, adjacent to a vaulted building (fb = frame brace, v = vaulted building).

Research in the region has shown that the basic unit of settlement was a stone platform delimited by retaining walls ranging from a single course to several meters in height and varying greatly in area. The majority can be identified as supporting extended households, although some supported civic-ceremonial structures. Others bear no superstructures or secondary platforms and may instead have been production loci. Both masonry and perishable pole-and-thatch buildings rested on these platforms (Fig 6). As noted, masonry buildings, especially those with vaulted roofs, can usually be discerned in the lidar imagery, but the foundations of perishable buildings are much more difficult to identify (Fig 6B and 6C). The numbers of buildings and secondary platforms on basal platforms are highly variable, but often they are arranged at right angles to each other, forming the sides of a rectangular courtyard.

Households resident on basal platforms dominate settlement across the north (e.g., Dzibilchaltun and Chunchucmil [45, 46]), but contrast with some areas of the central lowlands, where more often the basic unit is an elongated mound, often arranged with other such mounds to form the edges of a patio or courtyard group. (A similar contrast has been noted between the residential units of Caracol, Belize, also primarily basal platforms, and those of the central Peten [25].) Such mounds result from the collapse of buildings and often ground plans can only be determined by excavation. In contrast, the number and dimensions of rooms in a Puuc building are usually evident upon visual inspection, so that we have a detailed settlement record in those areas where we have carried out terrestrial survey. An important point is that in the central lowlands, building mounds are usually the basic unit of lidar analysis, whereas we use basal platform counts. Therefore, settlement statistics from the north are not easily compared to those from the south when demographic questions arise, since a basal platform is often the equivalent of a courtyard group, and thus a count of basal platforms substantially underestimates the number of actual buildings.

**Fig 7:**
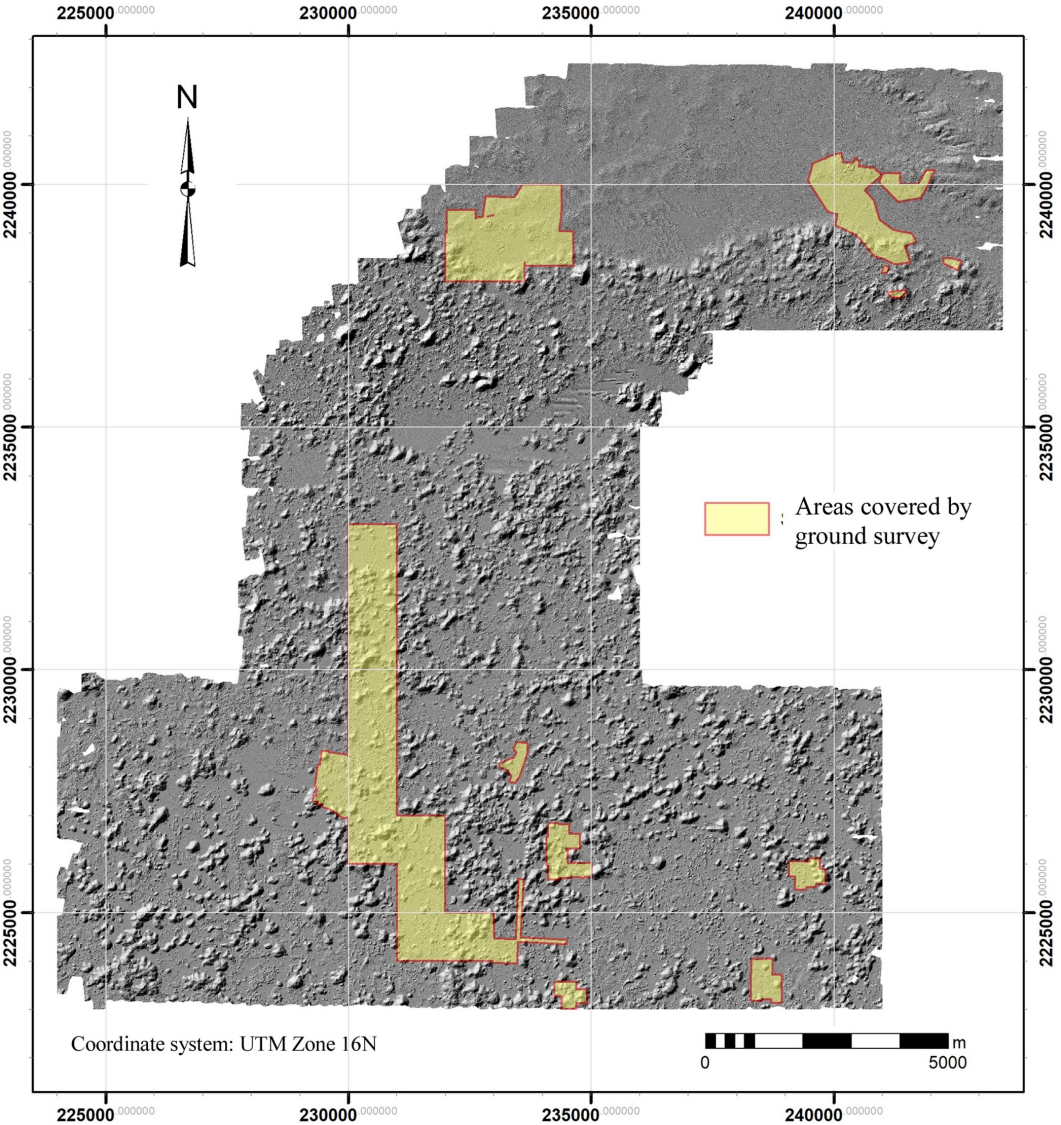
Areas covered by ground survey, 2000–2020. Total coverage is 21.4 km^2^.

We have currently registered 7902 basal structures by means of lidar imagery alone, as well as 1232 annular structures, of which more below. We have not yet systematically tried to identify buildings and secondary platforms except in those areas where we also have ground coverage (Fig 7). An example of one such survey area is the large site of Muluchtzekel, which provides some sense of the accuracy of tallies arrived at by visual inspection of the lidar data alone. At Muluchtzekel, 418 basal structures were identified in the imagery prior to ground survey (a further 237 lie outside the limits of our ground survey and await future verification). We have intentionally not registered secondary platforms and buildings at this stage of our analysis.

During ground survey of this area, a total of 1781 structures of all types were registered: 524 basal platforms, 1021 perishable and masonry buildings, 46 annular structures, and 190 secondary platforms. Thus, approximately 80% (418 of 524) of the basal platforms could be seen in the lidar, while 106 (20%) could not. Of the 106 that were not identified, 81 (76%) were small platforms lacking any building foundations. Of the remaining 25, 14 had a single frame brace, 8 had two, and 3 had three foundations, 39 buildings in all. Only one was a possible masonry building. In contrast, the 418 basal platforms visible in the lidar supported 874 buildings, versus the 39 buildings on the 106 platforms that were not visible, a ratio of 95.7% to 4.3%. (In addition to these 913 buildings, a further 108 were basal buildings, resting directly on the ground.) Thus, while a significant number of platforms could not be identified in the lidar, nearly all platforms supporting buildings were. Put another way, while we may not be able to identify all platforms and buildings in the imagery, we can be confident of identifying virtually all residential platforms.

**Fig 8:**
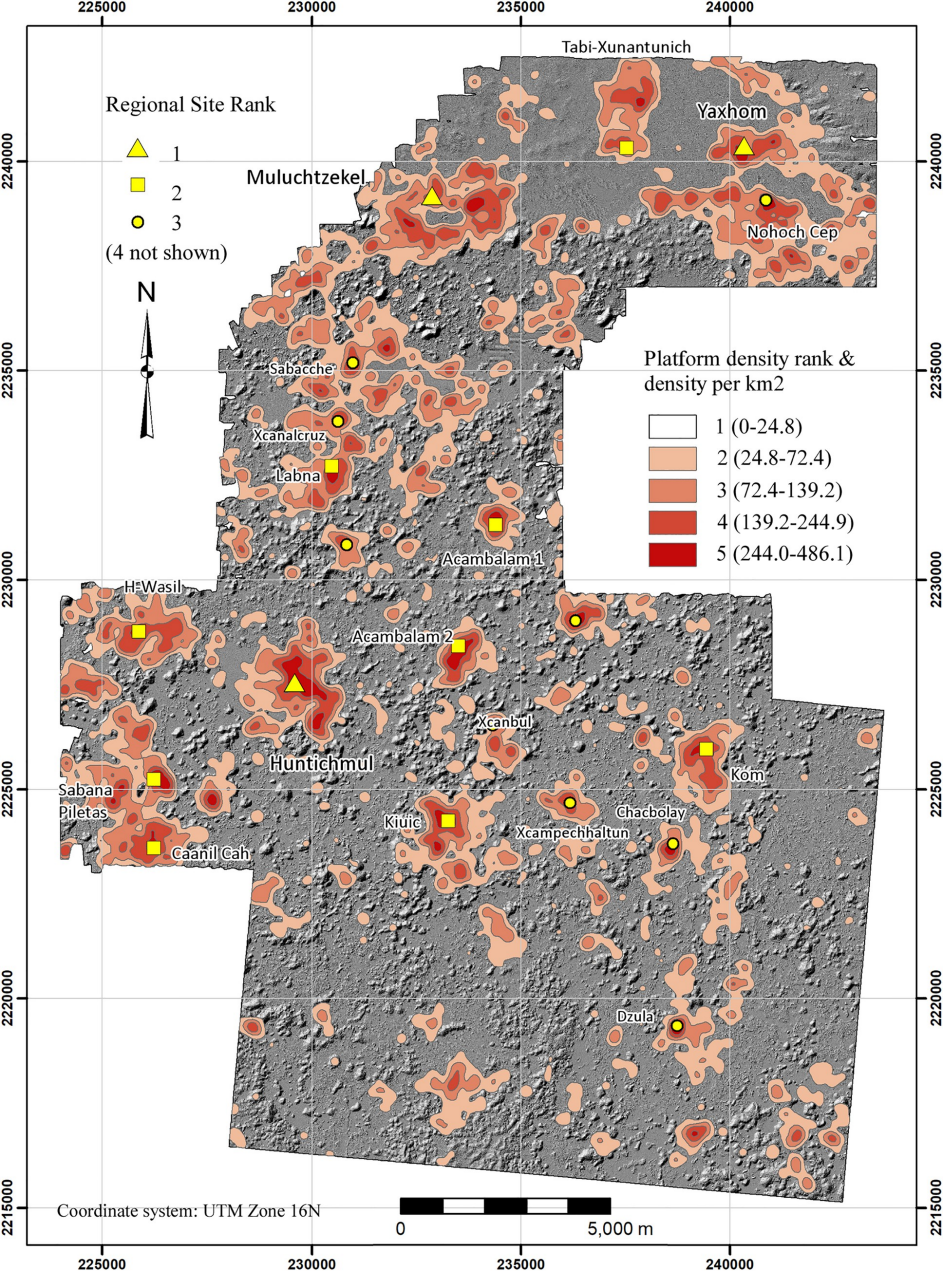
Basal structure density sectors within the NCALM and Alianza lidar samples. Note that Sector 1 is not colored.

We used the ArcGIS Kernel point density function, using a 250-m search radius, to approximate settlement density within the regional survey zone. Experimentation with search radii showed that 250 provided the best balance between resolution of settlement limits and connectivity. Lower values tended to isolate individual features while higher values overextended site limits and decreased site resolution. The resultant density gradient was then subdivided into five intervals defined by Jenks Natural Breaks to suggest community limits (Fig 8, Table 4). Although more intervals could have been used, five provided settlement categories that could be intuitively understood: 1) nearly vacant hinterlands, 2) rural hinterlands, 3) suburban, 4) urban, 5) site center. Intervals were then converted to polygons so that their areas could be measured and topological relations with other features assessed. One problem with the method is that occasionally false density isopleths are produced because of trends in the surrounding points. To remedy this problem, we calculated the actual structure density within each, using the ArcGIS spatial join function, and merged those whose actual density was less than the nominal value of the isopleth with the next lowest interval. Perhaps the most striking result is the high degree of population clustering. Sector 1, the largest structure density category by area, encompasses over 60% of the sample realm but is also the most lightly settled, containing only 393 platforms or 5% of the total. In contrast, the two highest density sectors (4 and 5) together account for 41.4% of the platforms in only 5.6% of the sample realm by area.

**Table 4:** Structure density sectors (see Fig 8).

Sector	Density Range	Sector Area (km^2^)	% Area	# Structures	Structure Density	% Structures
1	0–21.5	150.08	63.3%	393	2.6	5.0%
2	21.5–68.7	48.03	20.2%	1638	34.1	20.7%
3	68.7–137.5	25.76	10.9%	2658	103.2	33.6%
4	137.5–244.9	10.89	4.6%	2247	206.3	28.4%
5	244.9–547.7	2.48	1.0%	966	389.8	12.2%
**Total**		**237.23**		**7902**	**33.3**	

These results confirm the observations of Gallareta and May during their Labna-Huntichmul-Kiuic transect survey as to the relative scarcity of permanent intersite settlement [44, 47]. They further contended that platforms in the hinterlands are marked by the near absence of frame braces, *metates* (stone grinding basins), and *chultuns* (cisterns), an observation echoing Tourtellot et al. [48] concerning platforms near the limits of Sayil. To test this proposition, the 393 platforms of Sector 1 were inspected for *chultuns*. A total of 82 (20.9%) were positive, although 12 of these were tentative. Of these, 42 were within 100 m of a Sector 2 isopleth, and so were on the fringes of denser settlement. Thus, only 40 of the 393 Sector 1 structures possessed *chultuns*, were essentially isolated households, and were presumably occupied permanently.

**Fig 9:**
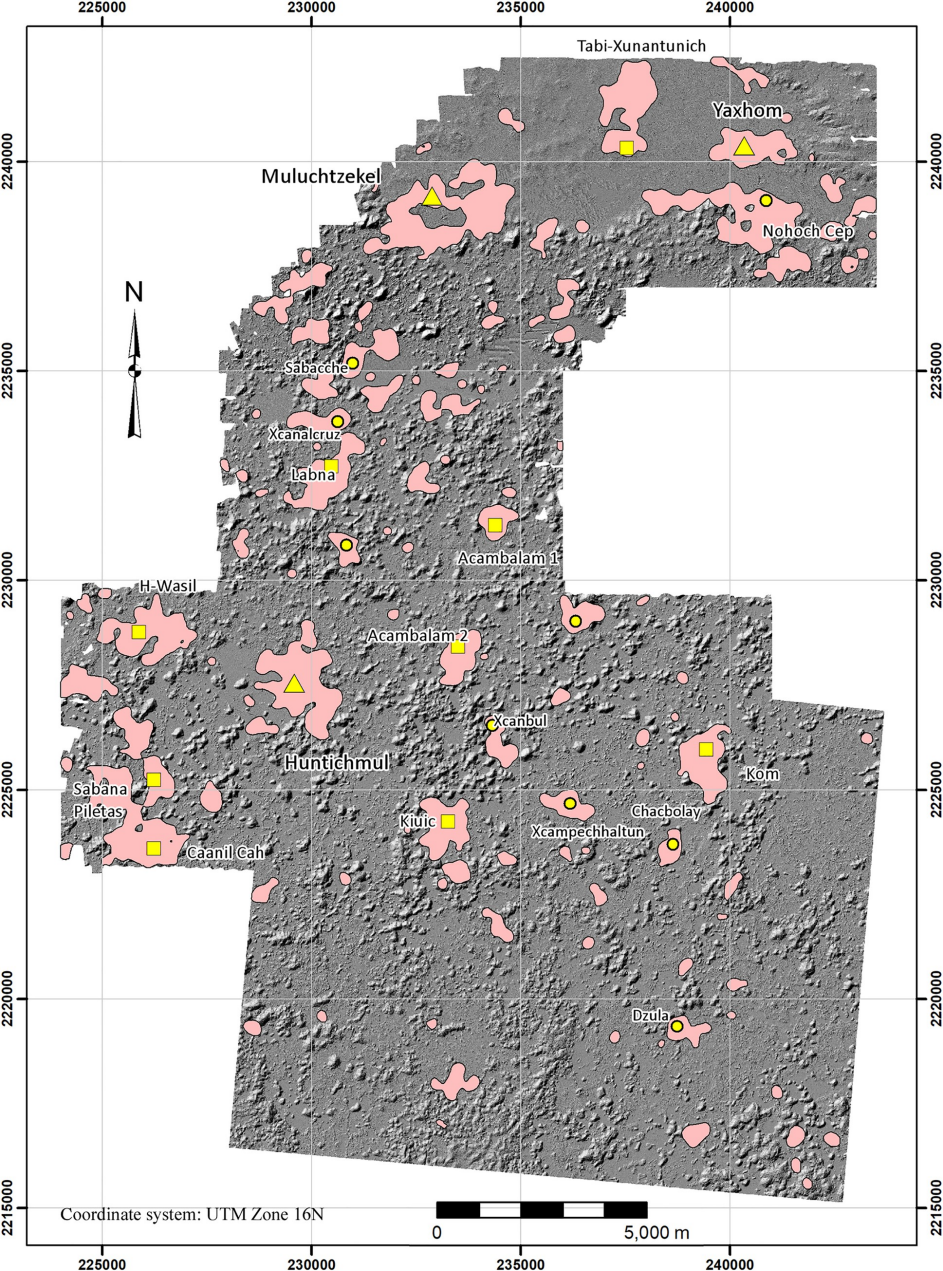
Major site cores within the lidar sample (merged structure density sectors 3–5).

If the top three density ranges (Sectors 3–5) are merged, the resultant isopleths correspond fairly well with known sites and generally clarify the separations between them (Fig 9). We define these areas as site cores, and the statistics associated with the ten largest are presented in Table 5. Site cores are only occasionally ideal concentric rings of population density. The majority, such as H-Wasil, Sabana Piletas, Muluchtzekel, and several others, have multiple nodes indicating less focused distributions, often representing clusters of civic architecture. In some cases, amorphous distributions extend between multiple civic cores: one of these extends from Labna north to Cabeza de Serpiente, another lies near the southwest extreme between Caanil Cah and Sabana Piletas.

**Table 5:** Site core* statistics of the ten largest communities.

Site	Area (km^2^)	Platform Counts	Platform Density
*Greater Yaxhom	5.740	820	142.9
Sabana Piletas-Caanil Cah	4.16	601	144.4
Muluchtzekel	3.91	574	146.9
Huntichmul	2.88	535	185.8
Labna-Xcanalcruz	2.18	320	146.7
H-Wasil	1.97	288	146.2
Tabi-Xunantunich	2.04	279	137.0
Kiuic	1.25	218	174.1
Kom	1.41	210	149.4
Acambalam II/III	0.91	186	204.2
**Total**	**20.707**	**3211**	**155.1**

* Site cores represent the fused platform density sectors 3–5 from Table 4 and are conservative estimates excluding the peripheries. Greater Yaxhom is treated somewhat differently for reasons detailed in the text.

The most aberrant is Yaxhom. Dunning [49] identified Yaxhom and its neighbors as a conurbation, that is, a complex of sites forming a larger whole. Indeed, several distinct site cores are arranged around the rim of the eastern Yaxhom *planada* consistent with Dunning’s characterization, but inclusion of Density Sector 2 connects them via a continuous U-shaped sprawl of lower density settlement, suggesting it was a single community. A common identity is reinforced by the central Aguada Xpotoit and the causeway system radiating from the Yaxhom civic cluster. Grouping these site cores brings the total area to 5.74 km^2^, substantially larger than any other site. If the periurban Sector 2 fringe is included, the expanse increases to 11.77 km^2^. A total of 820 platforms lies within the various site cores of greater Yaxhom, rising to 1022 if Sector 2 is included. Regardless of how Yaxhom is defined, it is clearly distinct from Muluchtzekel, a large center that dominates the western edge of the *planada* region. Another population agglomeration, encompassing the sites of Nueva Tabi and Xunantunich (in reality a single settlement sprawl), dominates the northern edge and is separated from both sites by relatively vacant lands.

### The Puuc landscape

**Fig 10:**
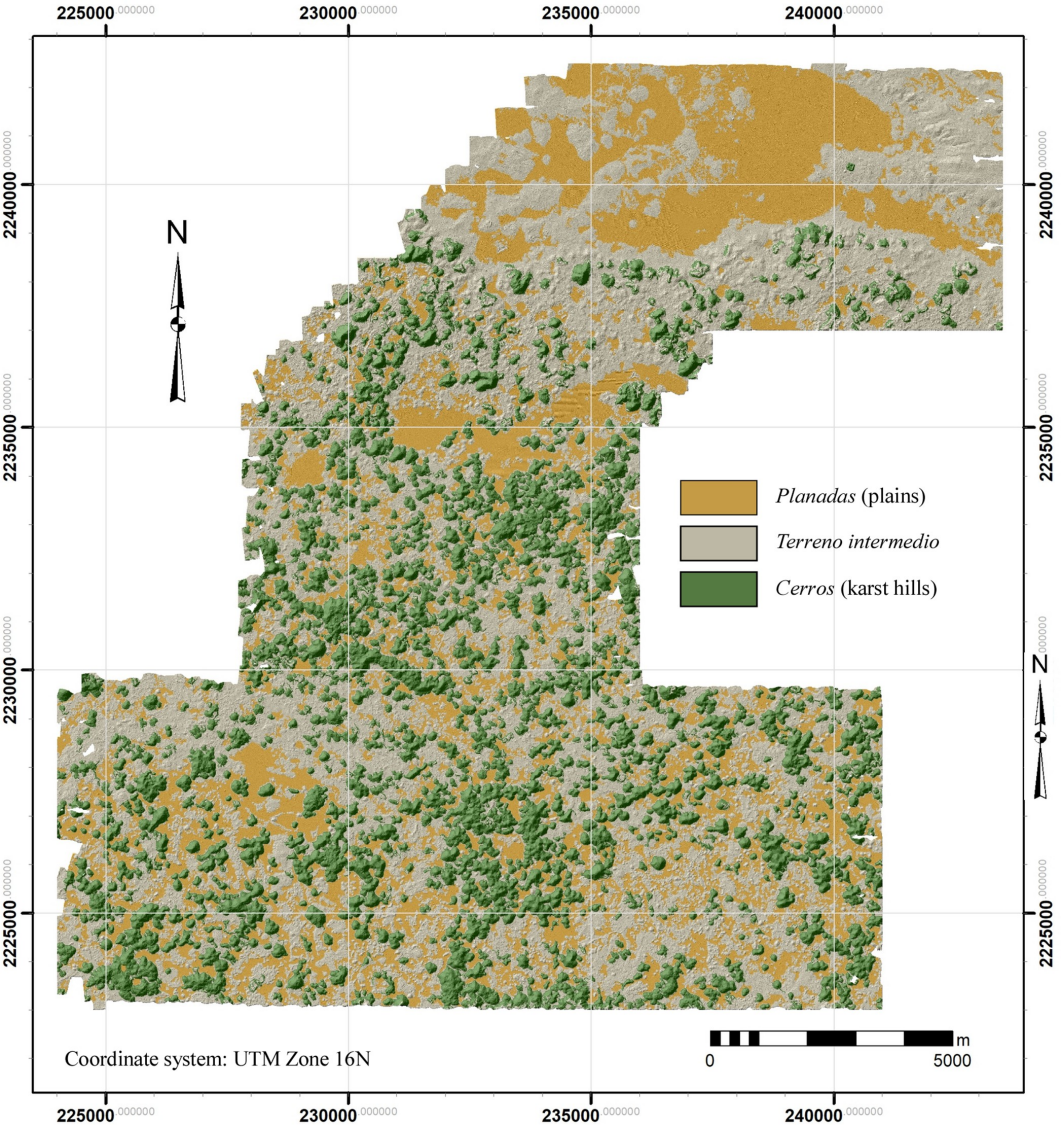
Map of the major classified landforms within the NCALM lidar sample.

Although certain aspects of the relation between landscape and settlement are immediately apparent–two of the three largest sites of the sample are to be found in the VSE–human utilization of the landscape can be studied in greater detail by classifying regional landforms by means of GIS analysis. Herein we distinguish three landforms, each present in varying proportions in the two major physiographic zones, the VSE and the DBC: 1) *planada* (plains or flats), 2) *terreno intermedio* (slightly elevated areas of modest slope consisting of ridge, hillocks, and hummocks), and 3) *cerros* (hills 15 m or more above their base). The junctures between the three classes are relatively abrupt, allowing them to be classified using GIS software (Fig 10).

*Planadas* were delimited by means of slope, since elevation often rises slightly across the large *planadas*, precluding the use of elevation isopleths. We found a slope of about 3% (1.7 degrees) or less yielded results which coincided nicely with the observed limits of certain *planadas*. Some *planada* polygons required manual adjustment because they were divided by modern roads and spoil banks, but in the interior regions very little refinement was needed. *Planadas* with an area of less than 1 or .7 ha (see below) were classed as *terreno intermedio* since they were probably too small for *milpas* (cornfields). *Cerros* were also defined by slope and a minimum altitude of 15 m above the surrounding terrain. Experimentation determined that the base of a hill was usually defined by a slope of 12 degrees or more. Classification by slope yielded a series of “donuts” (because the summits or side peaks had lesser inclinations), but these were easily closed by using the ArcGIS Union geoprocessing tool with "Gaps Allowed" unchecked. ArcGIS zonal statistics were then extracted for each *cerro* polygon, providing minimum and maximum elevations that could then be used to calculate height. There are, to be sure, a number of cases where these criteria failed and had to be remedied manually, but generally the results were quite satisfactory. *Terreno intermedio* is the remaining area, those with slope values between 1.7–12 degrees but also small *planada* parcels. Overall, *planadas*, *terreno intermedio*, and *cerros* occupy 25.6%, 46.8%, and 27.6% respectively of our study region (Table 6).

**Table 6:** Land forms and settlement statistics.

Class	Area (km^2^)	% Total Area	Platforms	% Platforms in Class	Density
*planadas*	60.7	25.60%	299	3.78%	4.92
*terreno intermedio*	110.9	46.77%	5635	71.31%	50.79
*cerros*	65.6	27.63%	1968	24.91%	30.02
**Total**	**237.2**		**7902**		**33.31**

Terrain elevation ranges from 28–155 m above sea level within the 19.5 km north-south extension of our survey sample. Locally, however, only 17% of the peaks are more than 50 m above the surrounding *planadas*, and the highest rises less than 90 m. (Heights were determined by means of GIS zonal statistics for each *cerro* polygon.) The hills are also relatively small, with an average circumference about 1250 m, often making them easier to walk around than to climb. The *planadas* likewise are of relatively modest extent. Most could have been easily crossed in less than an hour and the largest in just a few. Conversely, the smaller size of such features means they are more numerous: nearly 900 peaks and several hundred *planadas* over 1 ha fall within our survey limits.

**Fig 11:**
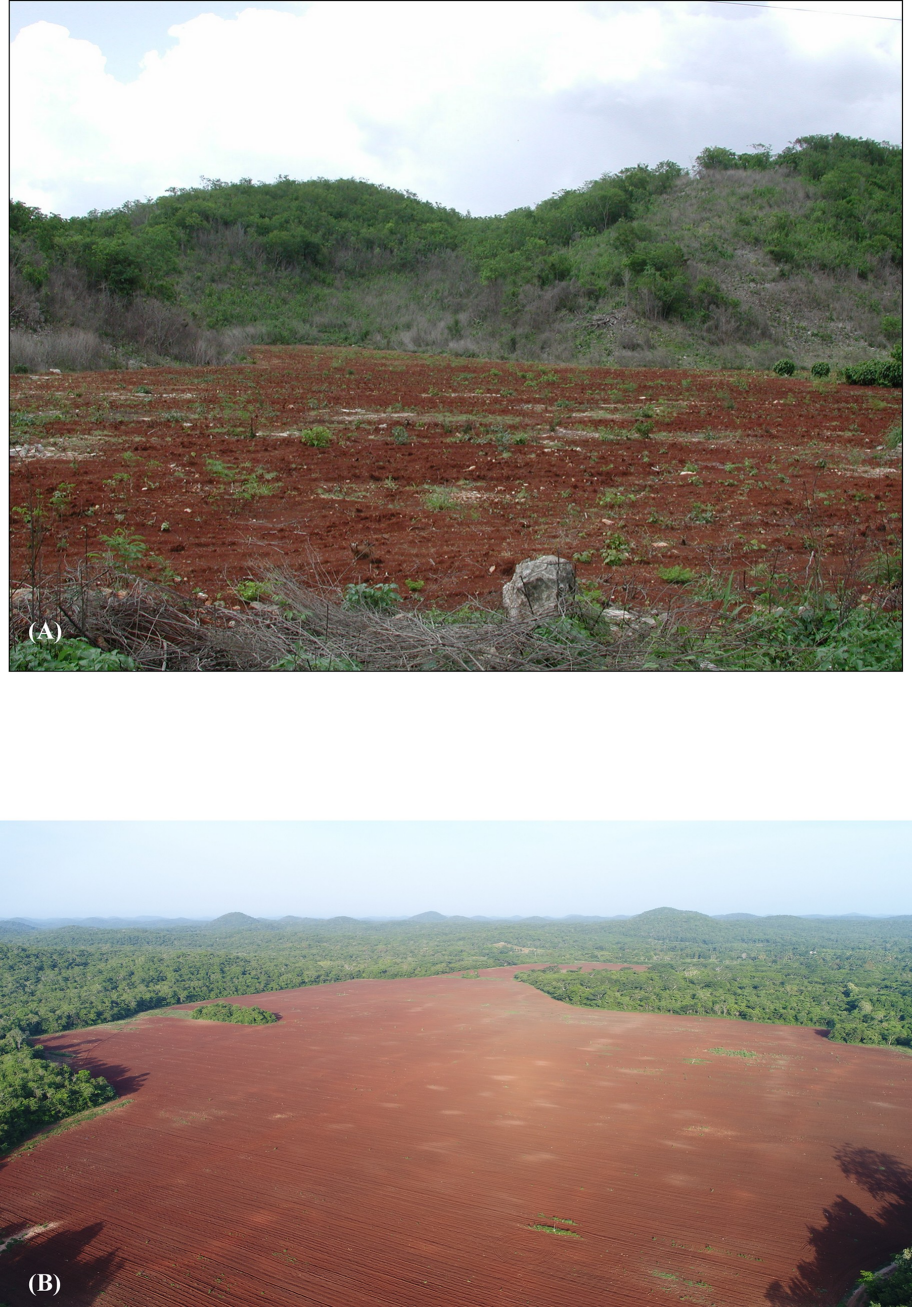
Puuc *planadas*. (A) A *planada* cleared for cultivation in the Distrito de Bolonchen. (B) A portion of the Muluchtzekel *planada*. The archaeological site is in the surrounding *terreno intermedio*.

In the past, the Puuc has sometimes been called the “granary of Yucatan” for its importance in selling maize to Merida during the colonial period [50–52], although these mostly refer to sales by towns at the foot of the Sierrita. GIS analysis of Puuc landforms can provide some insights into whether this may have been true during the Late-Terminal Classic. Puuc farmers today heavily favor cultivation in the *planadas*, where soils are deep and relatively fertile (Fig 11). The outstanding *planada* is the shallow basin known as the Valle de Yaxhom, with an approximate area of 15.14 km^2^ (13.46 km^2^ of which is within our survey bounds). This basin is exceptional in having deep deposits of fertile *ek luum* soils which today support intensive citrus cultivation. The Yaxhom site core overlooks this basin. Some modern cultivation is also carried out on the *terreno intermedio*, either because of an insufficiency of *planada* parcels or because certain insect pests are less bothersome in better-drained areas. The stony *tzekel* hill slope soils are a poor third option.

Our workers, who are mostly local subsistence farmers, generally cite a figure of around 1 ha as sufficient to feed a family for a year. This agrees well with field sizes recorded in the ethnographic and ethnohistorical literature (see [53] for a review of the literature). Steggerda [54], for instance, recorded an average *milpa* size of .99 ha based upon 638 *milpas* recorded over a five-year period across three towns in Yucatan, one at the foot of the Puuc. However, this included feed for domestic animals, estimated at about 44% of annual consumption, indicating about .7–1.0 hectares suffices for a single nuclear family.

In our study area, if parcels are limited to a minimum area of 1 ha, *planada* lands comprise a total of 60.754 km^2^ divided into 684 parcels of contiguous land, or 25.61% of the total study area. This was not distributed uniformly: the largest, a series of connected *planadas* covering much of the Valle de Yaxhom and environs, extends over 19.02 km^2^ (1902 ha), 31.30% of the total *planada* extent. When the second largest *planada* is added, a large expanse just to the south of the Valle de Yaxhom, the total reaches 39.16%. The five largest *planadas*, all substantially larger than the rest, constitute 47.84% of the total. In contrast, the median size of the 684 *planadas* is just 2.17 ha, strongly indicating a highly fractionated landscape.

These statistics allow a rough estimation of study area carrying capacity. If every 1+ ha *planada* parcel was under cultivation, approximately 6075 families could have been provided for. If a five-year fallow rotation schedule was in effect, with fields being cultivated for two years (the first newly cleared, the second replanted), two-sevenths (28.5%) of the planada land would be under cultivation, maximally supporting about 1735 families. A10-year fallow cycle could have been fed about 1010 families. If a value of 5 persons/nuclear family is used, a five-year fallow cycle could have maximally supported about 8675 people, for an overall density of 36.6 people/km^2^. If average *milpa* size is set to .7 ha, these figures can be multiplied by approximately 150%, since additional smaller parcels would be added to the total of cultivable land. In either case, the number of families is less than the number of basal structures (7902) and far less than the number of buildings they would have supported. Production must therefore have been increased by means of a reorganization of labor, by cultivation of additional lands, or both.

To estimate the potential of the *terreno intermedio*, nearly half the available terrain but also the most densely populated, we first approximated the size of a *solar* (house lot) by determining the average distance between platforms that were not isolated, defined as within 100 m of another platform, using the ArcGIS “Nearest” function. The average distance of the 6803 platforms so classified was 41.8 m, with a median value of 37.4 m, so a buffer 20 m in radius was calculated for each platform point as an approximation. We then excluded all of the land classified as site core (Sectors 3–5) and all of the “*solares*” in the remaining *terreno intermedio*, leaving a total area of 87.62 km^2^, or 79.5% of its total area. Since the *terreno intermedio* snakes along the edges of *cerros* and *planadas*, it was not possible to divide it into parcels as we did for *planadas*.

Using the parameters just mentioned, and estimating a productivity rate 80% that of *planadas*, an additional 7,030 families could have been sustained by annual cropping and 2,000 by a 5-year fallow rotation. Soils of this zone may have been more easily exhausted, however, so that a longer fallow estimate may be more appropriate. In sum, and recognizing the many caveats, approximately 3,740 (5-year fallow) -13,100 families (no fallow) could have been supported if both *planada* and *terreno intermedio* land was incorporated into a managed system. If the size of a nuclear family is estimated at 5 persons, this would translate to a population of between 18,700 to 65,500, or an average population density of 79–276 people per square kilometer. Again, if smaller *milpas* were the norm, these figures could be as much as 150% higher. Such figures, varying as they do by a factor of 3.5 or more, and subject to various other caveats, demonstrate the difficulty of approaching actual population levels by means of carrying capacity estimates alone.

Production may also have been enhanced through multicropping [49] and infrastructure (landesque) improvements. The existence of Puuc agricultural terracing has been speculated on for some time ([55]; see discussion in [4, 50]), but what Schmidt and others refer to are *nivelaciones* built on hill slopes, which are generally too small to have been significant agriculturally (see below, Fig 20). (*Nivelaciones* are platforms or terraces that grade into the hillside on one side and may or may not support additional superstructures.) Furthermore, *nivelaciones* are built with stone fill and often are closely associated with household platforms, indicating that they were areas for work or possibly storage.

**Fig 12:**
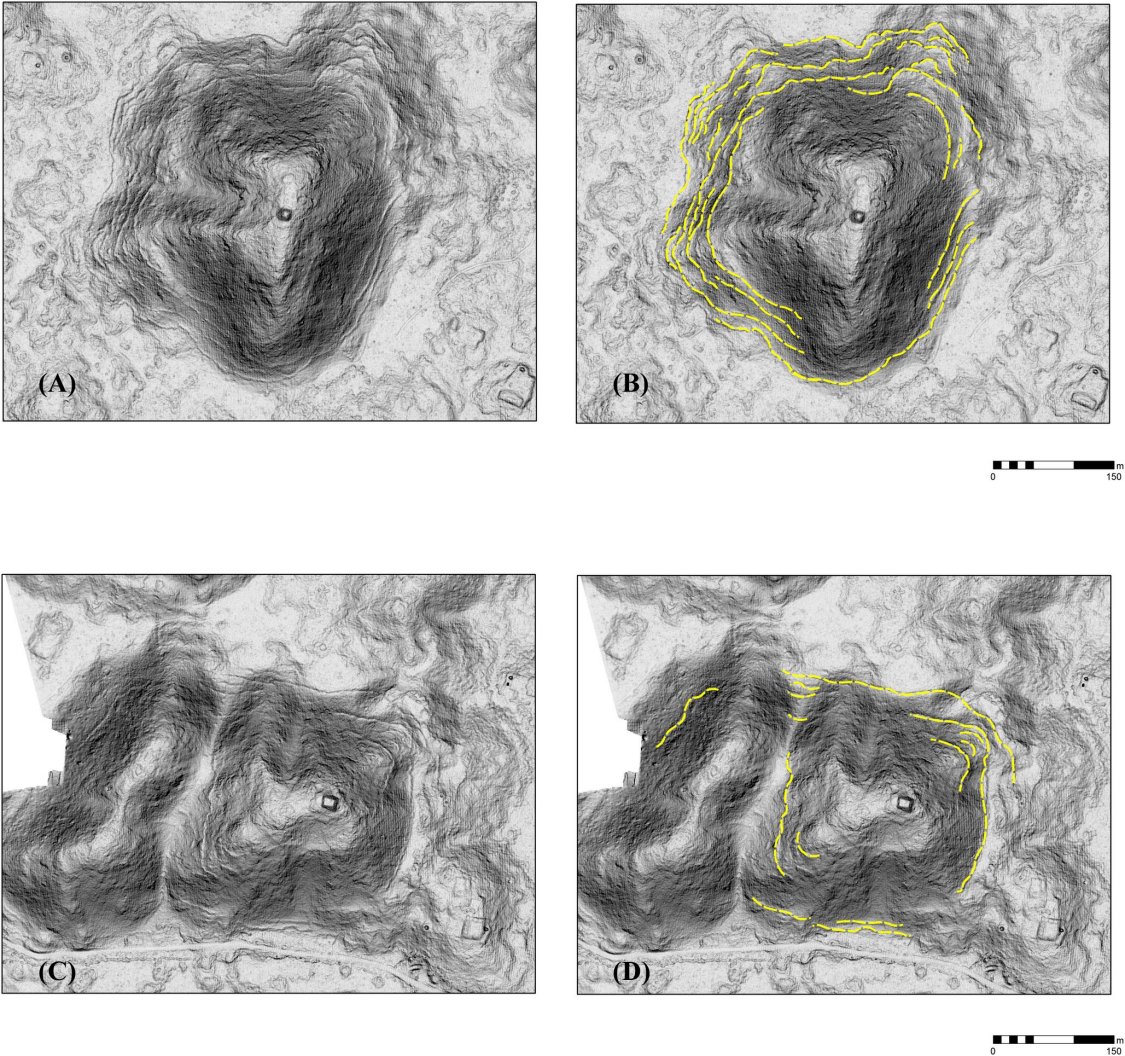
Terracing of Bolonchen cerros. (A, B) Cerro Ku Witz. (C, D) Hill west of Muluchtzekel.

Nevertheless, our lidar imagery did reveal the existence of true terraces on the slopes of a small number of *cerros*, two of which we inspected in 2019 and 2020 (Fig 12). The terraces made visible in the lidar imagery are long linear features that follow the contours of a given hill and were usually built in segments, rather than completely encircling a hill. The tallest hill bordering the Muluchtzekel *planada*, the *cerro* Ku Witz, happens to be terraced and in fact has the most terraces of any hill in our study sample. The Ku Witz terrace rings cover only the lower slopes and are 6–15 m apart, but the southern aspect has only a lower terrace. The terrace edges are of large unshaped boulders arranged to form retaining walls, behind which the terrain is generally flat and stone-free. Agricultural use seems the most parsimonious explanation.

Regionally, it is important to note that hill terracing was not widely practiced. We have identified only eight terraced hills among the many hundreds in our study region and, to date, none in the Alianza dataset. Second, the amount of terracing varies dramatically among these eight hills. Some have only one short segment, while the *cerro* Ku Witz has over twice as much terracing as its nearest neighbor. In all, the terraces total 10.59 km in length. The fact that they are confined to just a very few hills and only to the lower slopes, and that their total length is modest, does not suggest that agriculture was being intensified dramatically in this manner.

Furthermore, in general terraced hills do not seem to correlate with large sites. The exception is Muluchtzekel, which has the two most extensive terrace systems, including the *cerro* Ku Witz, yet in neither case does an elaborate elite household cap the summit. Both have at their peaks a substantial platform which may have supported a vaulted building, but both lack *chultuns* and associated support housing. A few terraced hills also have platforms with vaulted architecture, so that it is more likely that they functioned as observation points. In fact, all of the terraced hills are among the 108 hills we have identified as possible lookout points because of their relative height with respect to neighboring hills. In other word, terracing was only carried out on the tallest and steepest hills, contrary to what Dunning [4] states as the expectations for terrace locations.

### Civic architecture

**Fig 13:**
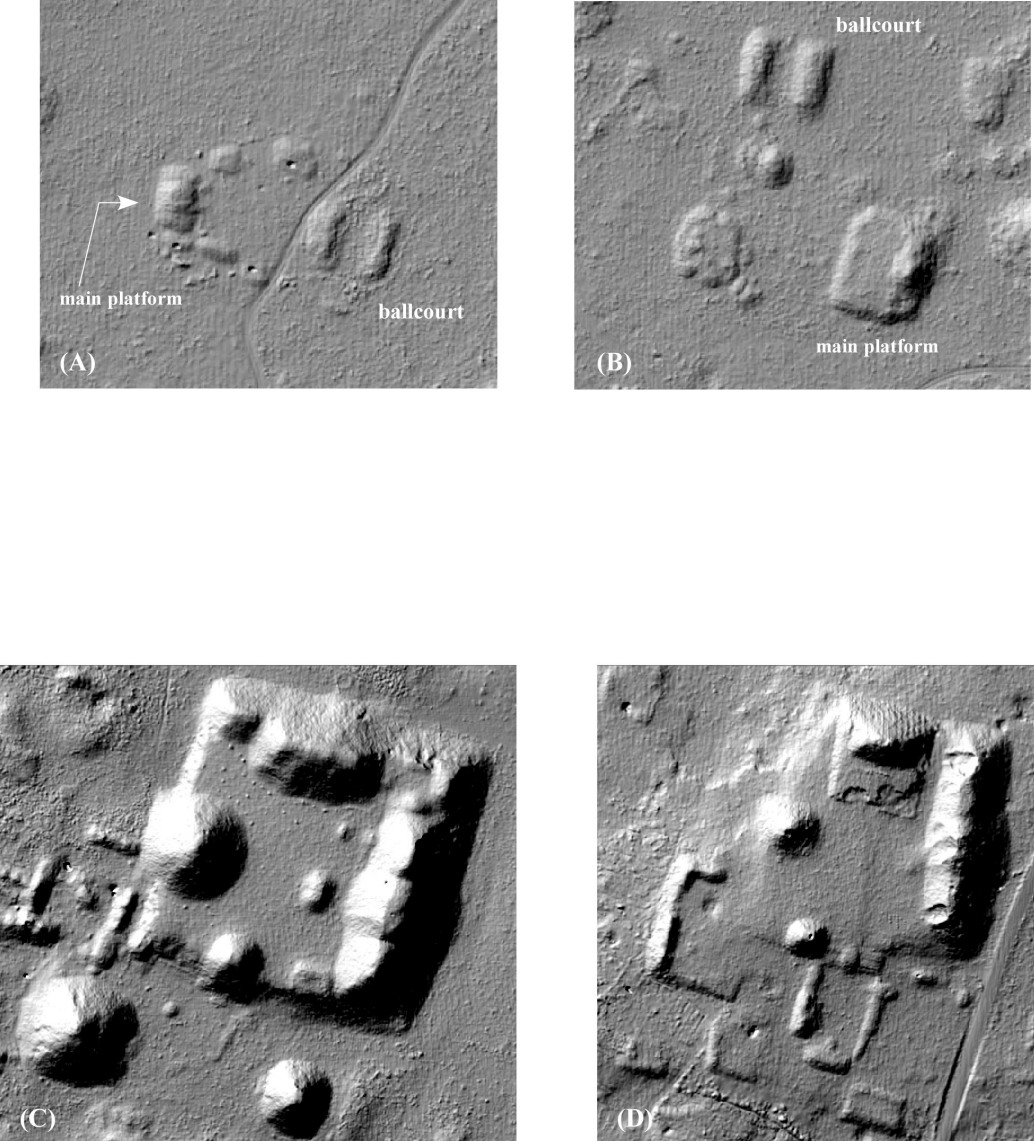
Middle Preclassic civic structures. (A) Paso del Macho, (B) a similar center near UTM N2233300E231700, Zone 16N, (C) the Yaxhom acropolis, (D) the Muluchtzekel acropolis. See also Fig 16B. All are to a common scale.

Lidar imagery reveals a range of public/civic architectural forms, a full accounting of which is beyond the scope of this paper. Here we treat examples from just two periods: early monumental architecture, from the latter half of the Middle Formative (ca. 700–450 BC), and the earliest civic forms of the Late Classic period, when the region began to experience a demographic boom, ca. AD 600–750. We have identified two types of Formative civic complexes. The first is exemplified by the small site of Paso del Macho, discovered in 2001 by Gallareta Negrón and Ramón Carillo Sánchez, test excavated by Gallareta in 2002, and more recently extensively excavated by Evan Parker (Fig 13A and 13B). The center consists of a small basal platform supporting a secondary mound along its rear edge. Two low mounds extending at right angles to the main platform define a plaza, at the opposite end of which is a ballcourt. As we have noted elsewhere [56], this arrangement bears a strong resemblance to Middle Preclassic civic complexes from northwest Yucatan, such as Benatunas [57]. The few known examples identified in the lidar are all within the DBC.

Much more substantial are a series of four Middle Formative acropolises, all except one within the Valle de Yaxhom (Fig 13C and 13D), the exception being a newly recognized example in the Alianza dataset, Uchbenmul. Three of these we identified first in the lidar imagery, and three have been test-pitted. The acropolises share a general orientation and layout, very similar to the acropolis at Xocnaceh located along the base of the Sierrita de Ticul [58]. They are the largest mounds in our study area (up to 145 m square in the case of Yaxhom), have a tall, elongated mound along the east side with three peaks (at the extremes and in the center), another tall mound along the north edge, less elongated but capable of supporting multiple rooms, and finally mounds along the south and west sides which probably supported only a single room. (The Uchbenmul acropolis is mirrored north-to-south.) No foundations survive on these mounds, however, and Late Classic cladding stones are nearly absent (in some cases the mounds did experience modest refurbishment during the Late-Terminal Classic). They may be the regional equivalents of E-groups, an early form of monumental architecture best known from the central Maya lowlands continuing into the Late Classic. These bespeak polities of a different order than the smaller centers of the DBC, probably financed by the extensive agricultural *planadas* they looked upon.

**Fig 14:**
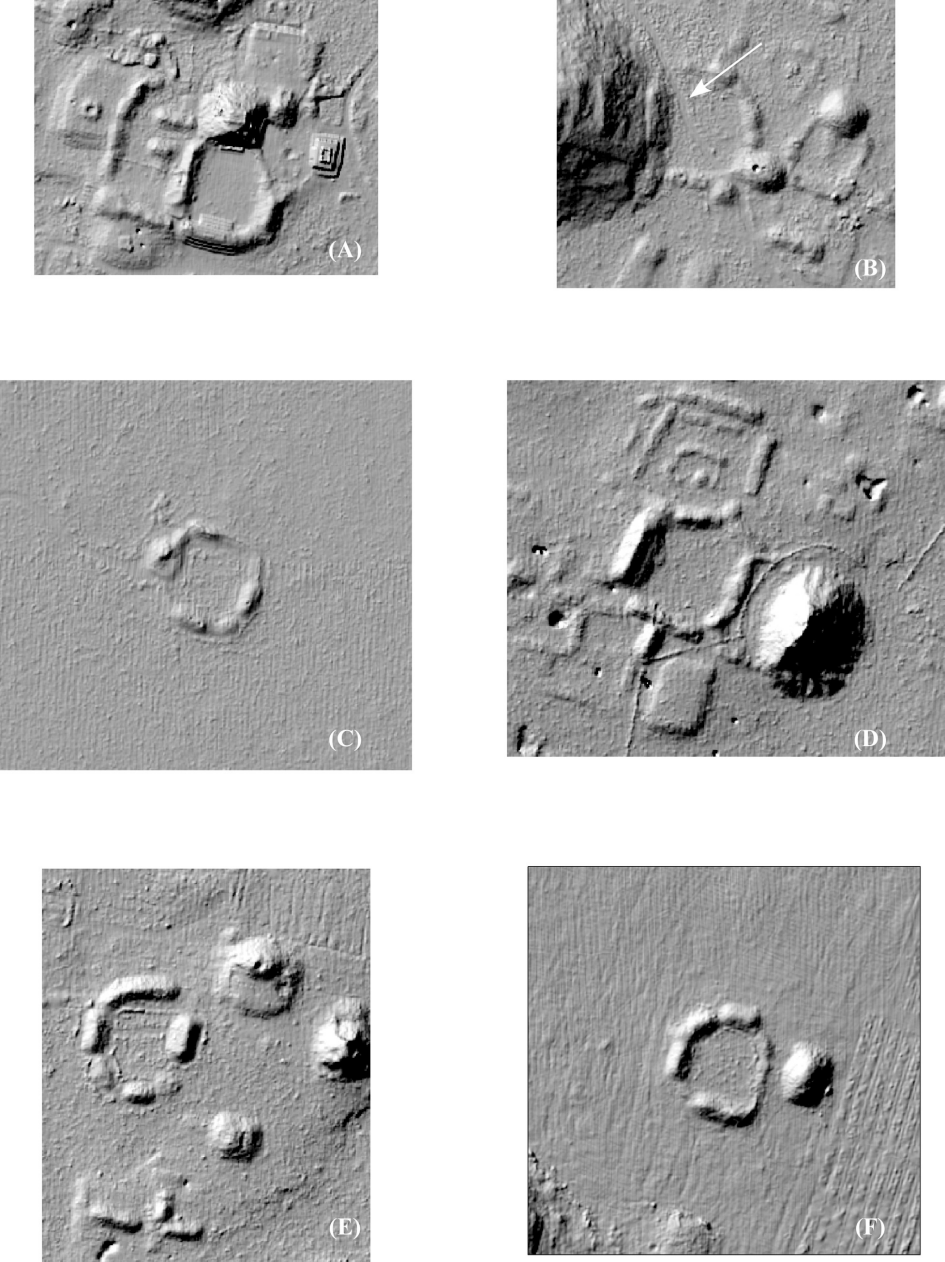
Examples of Early Puuc Civic Complexes (EPCCs). (A) Kiuic. (B) Huntichmul (1 of 2). (C) EPCC-J4870E1430. (D) Tabi-Xunantunich. (E) EPCC-J9215E9045, (F) EPCC-J6005E6160. All are to a common scale.

Regarding the Late Classic, in previous work we have identified an architectural complex which we refer to as the Early Puuc Civic Complex (EPCC) (Fig 14; [59]). The EPCC is quite different from other regional elite architectural complexes in that instead of being built on a platform, it consists of several buildings enclosing an interior plaza often built at ground level. EPCCs are either rectangular or sub-rectangular and often have connecting platforms crossed by means of ramps rather than stairs. One constant is a long hall with multiple entrances and a broad fronting stairway. The hall is usually just a single room, although sometimes small end rooms are attached. This is almost certainly what is called a *popol nah*, or council house [60]. Occasionally a *picota* (an uncarved columnar monument) was placed at the center of the patio. Buildings have little or no carved stone decoration apart from vertically faced moldings (often the medial molding is “broken”), and vaults are sometimes absent, or may be of slabs or small veneer stones. In short, the architecture is typical of Andrews’s [6, 7] Early Puuc Style.

Interestingly, such complexes are often linked to later palaces or other concentrations of public architecture by *sacbes* (causeways), as is the case at Labna, Kom, and Kiuic. Fig 23 shows this pattern at the newly identified site of Caanil Cah, near the southwest corner of our sample. One *sacbe* leads from a peripheral group located on a hill to the edge of a *planada* about 325 m distant, the purpose of which is unclear (not visible in Fig 23). The other two extend from an EPCC to what appear to be palaces or very large elite vaulted buildings. During a brief field reconnaissance, we verified these were stylistically later than the EPCC. Often earlier EPCCs seem to have been kept up and occupied even after larger palaces were built and in some cases were renovated by the addition of later rooms and/or buildings. Sometimes small stepped pyramids were built over one of the original vaulted buildings [61]. The intent was to legitimate later seats of power by means of a physical link to these earlier expressions of civic authority.

**Fig 15:**
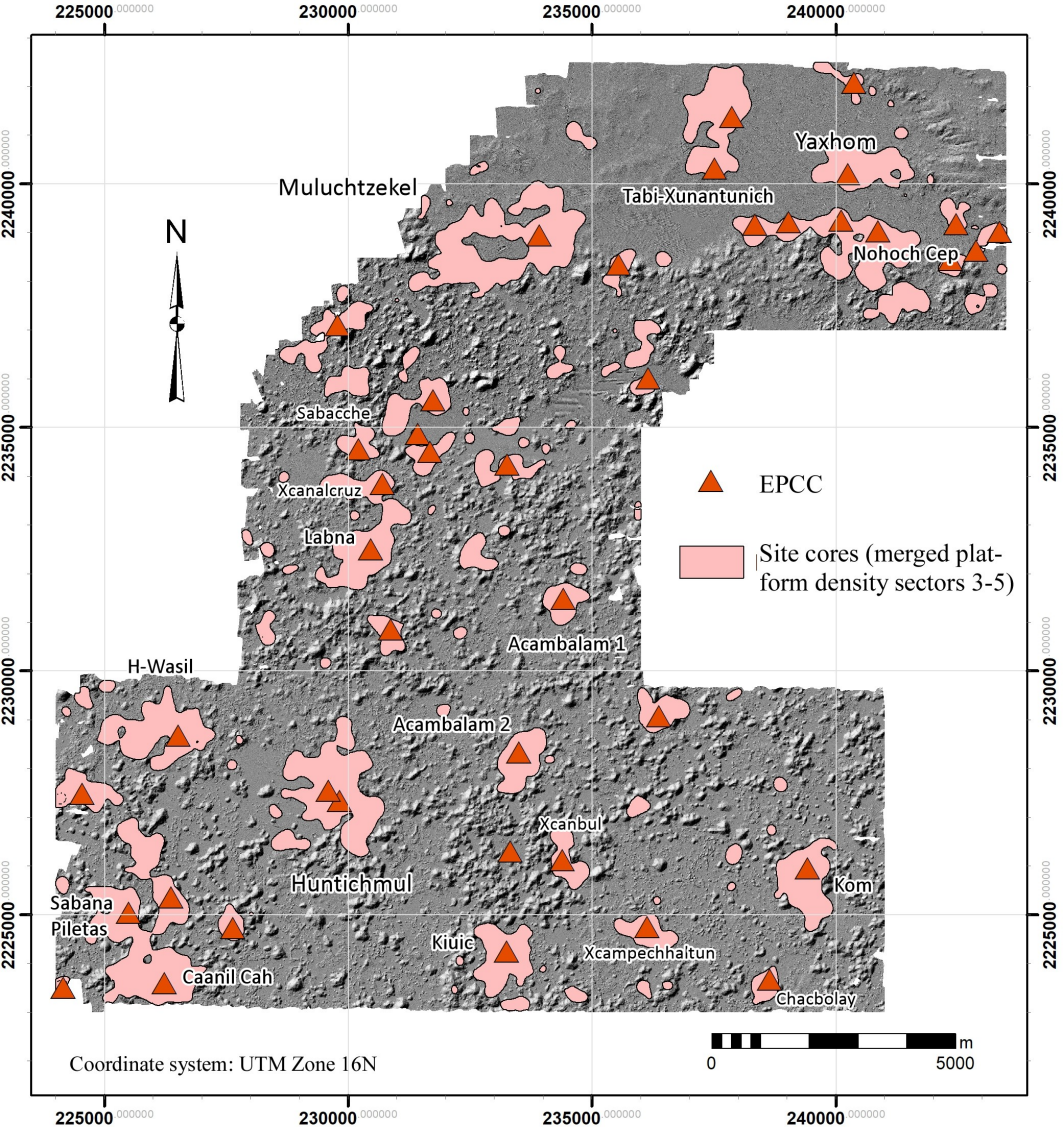
Locations of Early Puuc Civic Complexes (EPCCs) within the lidar sample.

Forty-two EPCCs have been identified so far in the NCALM imagery, ten of which we have visited, mapped, or excavated (Fig 15). All consistently display Early Puuc architecture, including a *popol na*. Every major center in the study region has an EPCC, sometimes two, but since there are more EPCCs than major centers, over time some EPCCs were probably absorbed by more successful and expansive neighbors. Thus, if we could better control for chronology during the Late-Terminal Classic, the pattern of nucleation observed in the distribution of settlement might be even more pronounced. We have identified only five EPCCs in the Alianza dataset, a further indication of the diminution of settlement intensity to the south and southeast.

EPCCs also mark ownership or administrative authority over land differently than in later periods. Later palaces tend to be built on low eminences, at the base of hills, or sometimes even on top of *cerros*, such as at Huntichmul. In contrast, and despite the generally low level of settlement in the *planadas*, 55% of the 42 EPCCs are within *planadas* and another 15% are at their edges (e.g., Fig 14C and 14F). The remaining 30% are located within the *terreno intermedio*, while none are known from *cerros*. A striking example of this distinctive settlement pattern is the several EPCCs along the interface between the Valle de Yaxhom and surrounding higher ground, probably a series of small polities, each with a claim to the immediately adjacent *planada*. Another example is the five EPCCs associated with the Sabacche *planada*. Three are positioned near the midpoint of major expanses within the *planada*, suggesting they too divided the optimal plots between them. Two further EPCCs are positioned at the north and south extremes of the western subdivision and are embedded within a larger community, while the first three EPCCs are isolated. (However, the eastern EPCC is closely associated with the largest *aguada* of the basin.)

To give an approximate idea of the territories surrounding each EPCC, we calculated Thiessen polygons around each example, recognizing the limits of the method (in cases with two EPCCs, we selected only one). Edge effects were significant in those EPCCs near the limits of the sample, leading to overestimates of their size. When these were eliminated, only 13 cases remained, but the results are illuminating. The average realm was only 5.2 km^2^ (median 4.9 km^2^), with a maximum of 12.5 km^2^ in the case of Huntichmul and less than a square kilometer around Sabacche. Although suffering from edge effects, clearly EPCC realms were especially small around the eastern end of the Valle de Yaxhom and the Sabacche *planada*. Another illustrative statistic is the distance to the nearest neighboring EPCC. Ignoring edge effects, the maximum distance was only 3.28 km and the mean distance was only 1.49 km (median 1.368 km), again pointing to the small size of these polities.

**Fig 16:**
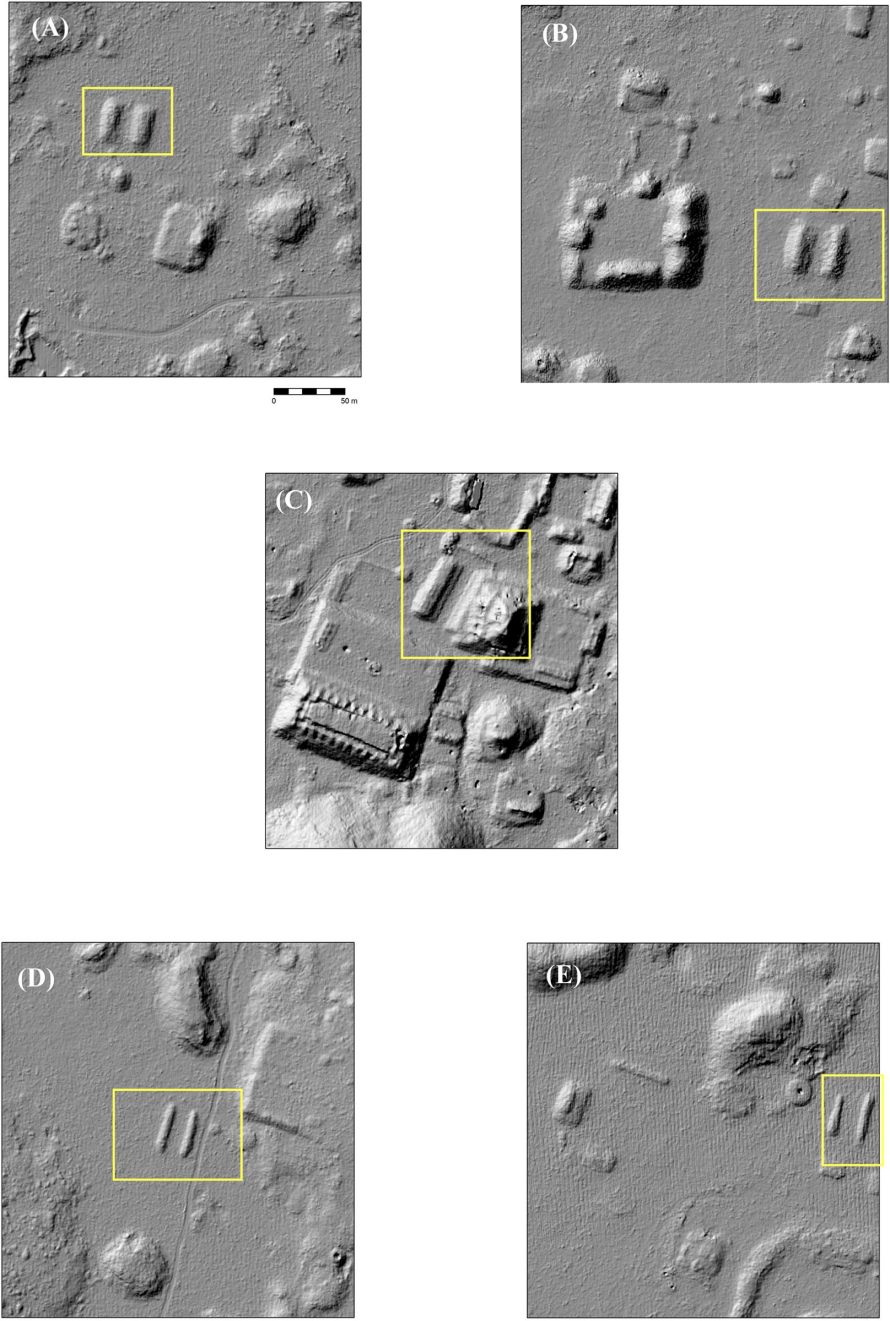
Ballcourts within the lidar sample. (A) a probable Middle Preclassic example near UTM N2233300E231700. (B) a newly identified acropolis, Uchbenmul, possibly dating to the Middle Preclassic. (C) Late-Terminal Classic period example from Sabana Piletas. (D, E) possible small ballcourts.

Finally, we have identified several ballcourts in the lidar imagery (Fig 16). Ballcourts are generally considered to be rare in the Puuc region [62], and our survey work has shown that some claims of Puuc ballcourts are unfounded, as for instance at Huntichmul. The Middle Formative ballcourt at Paso de Macho has already been mentioned, as has a possibly parallel site (Fig 13A and 13B), but another possibly early ballcourt lies next to the recently identified acropolis in the Alianza dataset mentioned above (Fig 16B). The date of the acropolis is currently unknown, but formally is likely to be Middle Formative, as thus may the ballcourt. Another substantial ballcourt, almost certainly Late-Terminal Classic in date, is located at the center of Sabana Piletas, near one of its palaces (Fig 16C). A final group of about 10 examples consists of two long, parallel mounds, oriented either NS or NE-SW (Fig 16D and 16E). Excavation will be necessary to determine whether these are in fact small ballcourts.

### Stone procurement and processing

**Fig 17:**
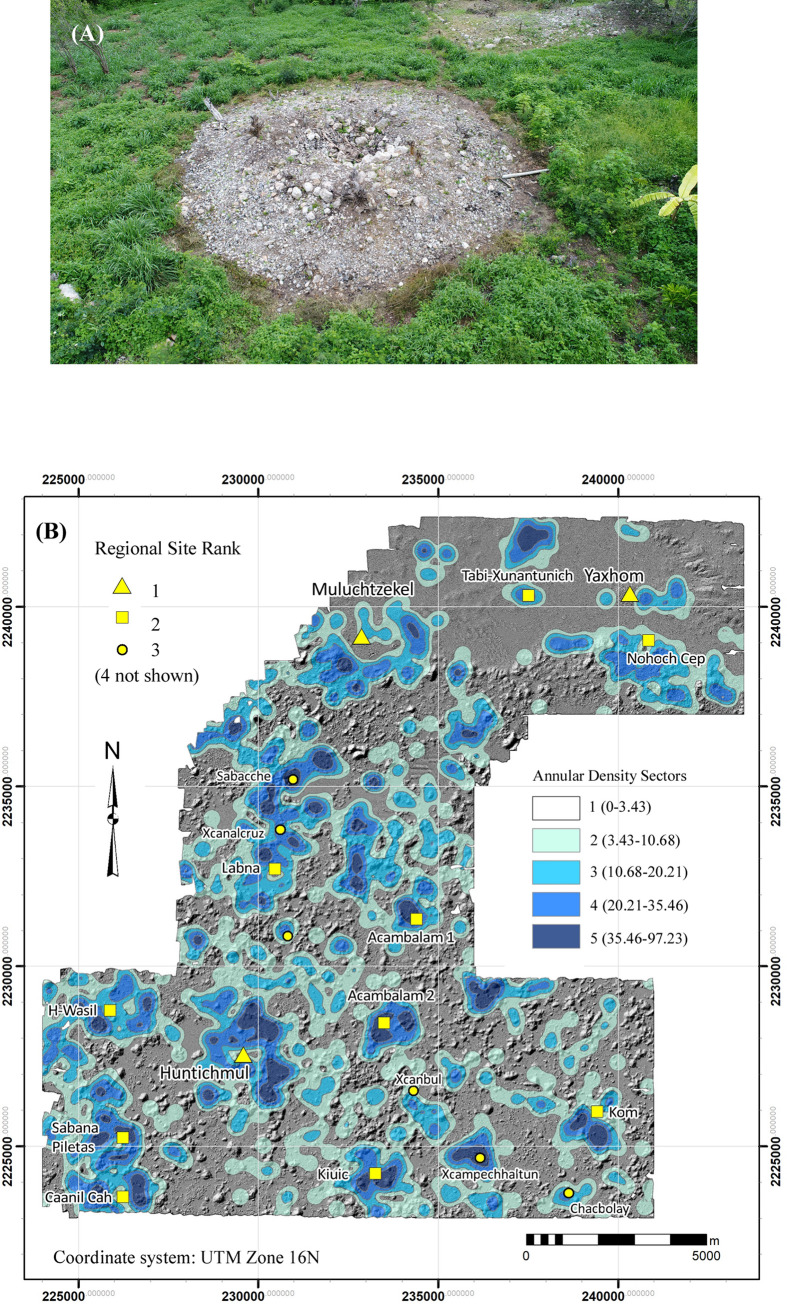
Annular structures. (A) An example of an annular structures. (B) density distribution of annular structures.

The frequency and quality of Puuc masonry construction attests to an important sector of the economy whose aspects remain poorly understood [37, 63, 64]. In the 1980s, mapping at the nearby site of Sayil had encountered 24 rubble mounds they dubbed “ring structures” [28], referred to as annular structures herein. These rubble rings, about 10 m or so in diameter and about 1–1.5 m tall, surround a central depression which often has signs of burning (Fig 17A), leading to speculation they may have been used to fire ceramics, make fertilizer [65], or to produce lime. Excavation of several of these by our project found almost no ceramics, leaving lime production as the most likely alternative. Experiments by Seligson, Gallareta, and May [64, 66] showed that annular structures were indeed efficient at producing lime, an essential ingredient in making mortar and stucco for construction, but also for the process of nixtamalization, the soaking of maize in lime water to soften kernels and enhance their nutrients.

Annular structures are quite visible in the lidar imagery, and to date we have identified 1232 of them (Fig 17B). Given the apparent scale of lime production, we might ask whether this was an urban activity, carried out in proximity to residences and building sites, or whether lime was produced in the countryside, nearer to raw material sources and fuel. Examining the distribution of annular structures with respect to our platform density sectors demonstrates a strong correlation between the two distributions (Table 7). Although 48.1% of the annular structures are located in the lowest two density intervals, the site core intervals, containing about 74% of the population, encompass 51.9% of the annulars. If they are looked at in terms of density, the hinterland (Sector 1) annular density is only about one-tenth the density of Sector 4, the densest, and overall annular density in the site cores (Sectors 3–5) is over five times that within the lowest two population isopleths.

**Table 7:** Annular counts/density in platform density sectors (see Table 4).

Platform Density Sector	Sector Area (km^2^)	% Area	# Annulars	Annular Density	% Total Annulars
1	150.08	63.3%	272	1.81	22.1%
2	48.03	20.2%	320	6.66	26.0%
3	25.76	10.9%	389	15.10	31.6%
4	10.89	4.6%	210	19.28	17.0%
5	2.48	1.0%	41	16.53	3.3%
**Totals**	**237.24**		**1232**	**5.19**	

With respect to our three classes of landforms, annular structures are found overwhelmingly on *terreno intermedio* (Table 8), with over 79% being present in this sector, vs. 16% on *cerros* and a small number on *planadas*. Even when corrected for area, the density of annular structures on *terreno intermedio* is still roughly three times that of the *cerros*. Interestingly, about a quarter of the annular structures on *terreno intermedio* were also within 20 m of the edge of a *cerro*, suggesting that was a favored spot for the manufacture of lime. Raw material could simply have been tumbled downhill and then broken up for processing. Others were closer to the edges of *planadas*, however, or were even within them, for reasons presently unclear.

**Table 8:** Landforms and annular structures.

Class	Area (km^2^)	% Total	Annular Strs.	% Annulars in Class	Density
*planadas*	60.73	25.60%	65	5.28%	1.07
terreno intermedio*	110.94	46.77%	979	79.46%	8.82
*cerros*	65.56	27.63%	188	15.26%	2.87
**Total**	**237.23**		**1232**		**5.19**

* Of those in the *terreno intermedio*, 138 are within 10 m of a *cerro* base, 248 are within 20 m (cumulative).

**Fig 18:**
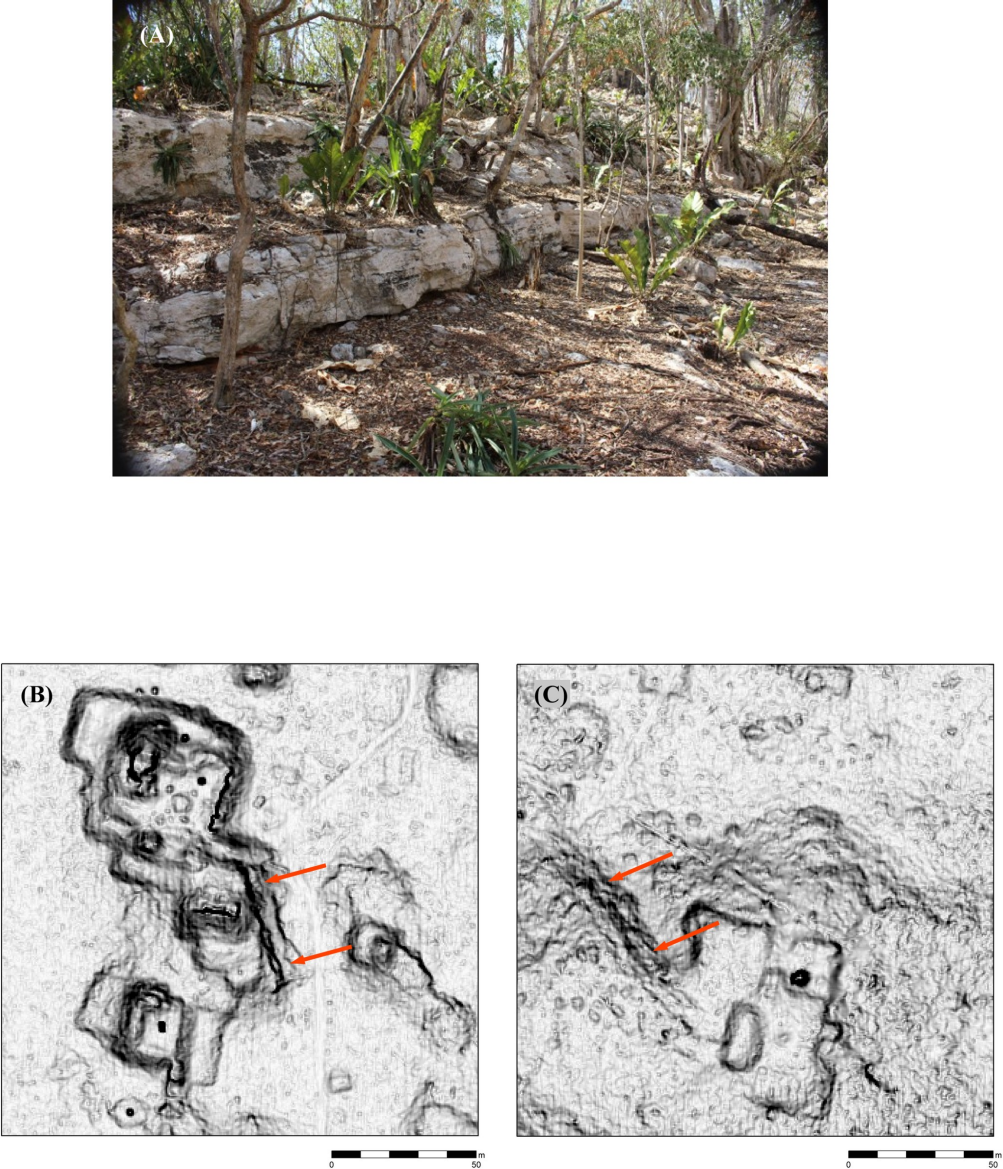
Evidence of stone quarrying visible in lidar imagery. (A) a section of the ledge quarry at Kom. (B) the Kom ledge quarry in the lidar imagery, marked with arrows. Note proximity to palace group. (C) a ledge quarry at Muluchtzekel, marked with arrows.

While the size of certain structures might suggest that enormous stone quarries would be found, that has not proved to be obvious. Stone was probably quarried in shallow pits in areas that were relatively flat (Fig 4) or by prizing stone from the ledges of hills and hummocks (Fig 18). Particularly notable ledge quarries can be found along the edges of the palace complexes at the site of Kom and Labna, where the relatively straight edges of the ledges indicate they are not natural. Another to the southeast of the major civic complex at Chacbolay is located adjacent to the largest annular structure at the site.

**Fig 19:**
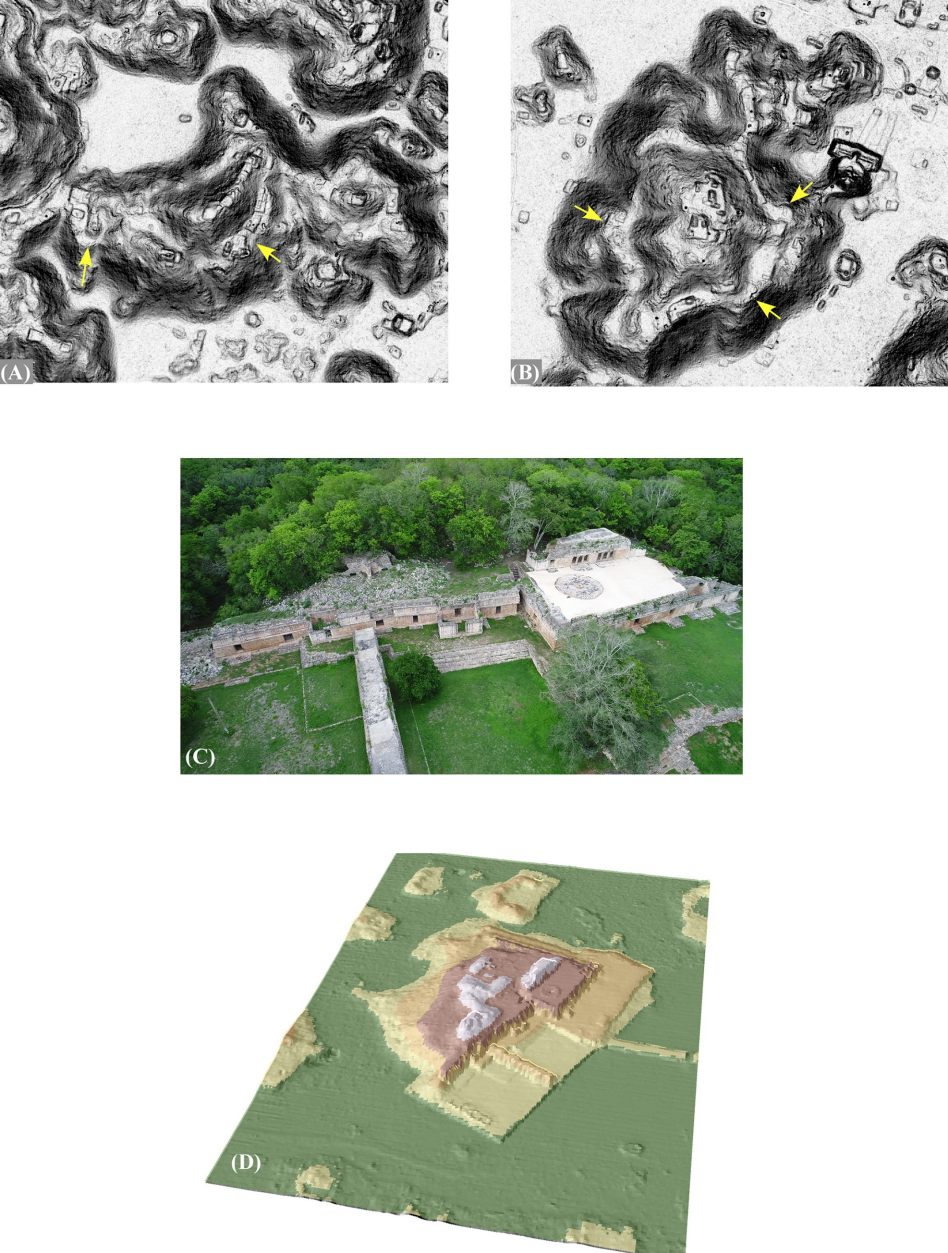
Modification of *cerros* (hills) by quarrying. (A, B) two *cerros* with terraces that may result from quarrying. (C) the Labna palace. (D) lidar imagery of the Labna palace, showing how the hill behind it was excavated for the building site.

Labna indicates the scale quarrying could assume (Fig 19C and 19D). It has long been noted that its palace was built against a hill, but a 3D lidar view strongly suggests that a large part of the hill was quarried away to make the building site and ended up as building fill. These sorts of operations would be largely invisible on the ground if the quarried material was recycled as construction fill. Elsewhere, several hills have ledges or landings supporting substantial platform mounds, often with vaulted buildings (Fig 19A and 19B). These appear to be too wide to be natural features and so may also result from hill quarrying.

### Use of the hills

**Fig 20:**
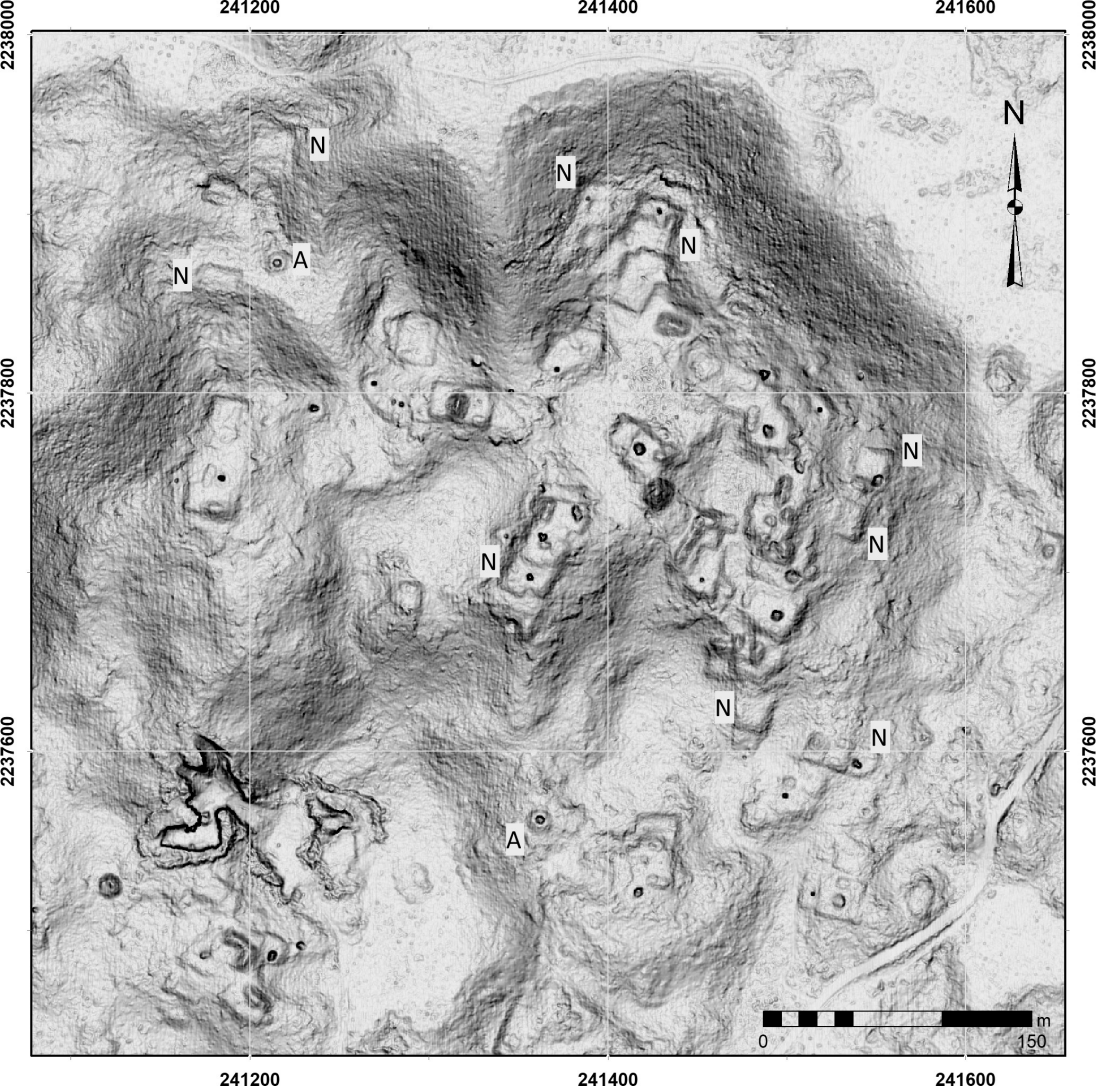
*Cerros industriales/residenciales*. Hills supporting possible corporate residential units involved in common production activities. N = nivelación, A = annular structure. Not all instances are labeled.

Investigators from Teobert Maler onward have noted the occasional placement of a vaulted building atop one of the Puuc *cerros*. Prior to lidar acquisition, full survey of hills by PARB personnel revealed that some of these hills also stood out for the density of their occupation. Some had not just a single vaulted building on their summit, but entire architectural complexes, such as the Escalera al Cielo excavated by Bey and colleagues [43]. Hilltops sometimes include multiple basal platforms, several vaulted residences, and small temples, together with perishable buildings serving a variety of functions, including housing, cooking, and storage. But such hills are also notable in having construction along their flanks and around their bases, usually less elaborate than the structures at the summit. In addition to residences, the flanks of hills often support *nivelaciones* perhaps used for some type of production, such as stone working [63, 67]. This clustering of occupation suggests that densely occupied hills can be considered as corporate groups of some sort, perhaps associated with particular parcels of farmland nearby or involvement in some type of production. For these reasons, we have dubbed them “*cerros residenciales*” or “*cerros industriales*” (Fig 20) whose economic focus in some cases may have shifted seasonally between cultivation, forest management, and stone processing.

Our analysis of such hills is ongoing, but the platform data and our classification of landforms can provide some preliminary insights. One question is the frequency of such hills. An initial hypothesis was that the larger *planadas* would have one or more associated *cerros residenciales* providing the workforce and management responsible for its cultivation. Our GIS analysis indicates that while about a quarter of the platforms are located on hills (Table 6), only 52.87% of the 892 *cerros* defined by GIS analysis were occupied, and only 11.7% had more than four buildings. Thus, *cerros residenciales/industriales* are rather infrequent. On the other hand, several of the highest structure density isopleths were located on hills, including 73 of the 78 isopleths with a density over 100. The isopleth with the highest density occurred on a hill near Sabana Piletas, enclosing 133 structures. Not surprisingly, most of the dense hilltop patches were also adjacent to *planadas*.

The locational constraints of *cerros industriales* are less clear, but if they were involved with stone working, it would be expected that several annular structures would be present among the other structures. Only 119 (13.41%) of the hill polygons contained an annular structure. All but 17 of these also contained a basal structure, but only 40 hill polygons had more than a single annular structure. A hill to the west of Caanil Cah had the most annular structures (10), with another 3 on an adjacent hill and several more on the *terreno intermedio* and *planada*. Given that the *planadas* around that site are relatively small, stone processing was probably its principal activity. Overall, *cerros industriales* appear to be infrequent, however.

### Water procurement

**Fig 21:**
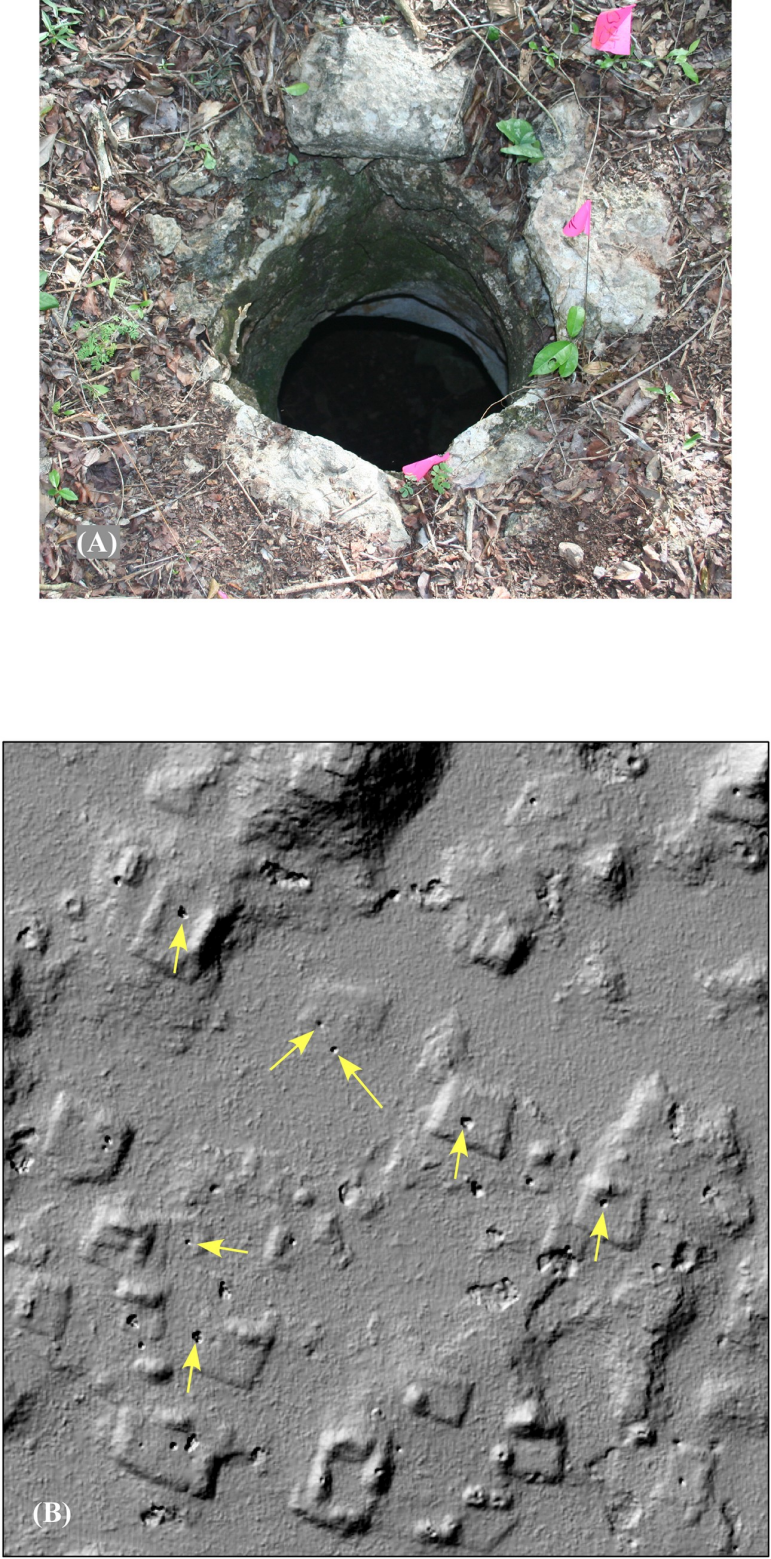
Water storage cisterns (*chultuns*). (A) *chultun* neck. (B) *chultun* openings visible in lidar. Larger pits are collapsed *chultuns*. Not all instances are marked with arrows.

Because geological uplift of the Puuc rendered the water table too deep to tap with premodern technology, and because surface bodies of water and deep caves were virtually non-existent, residents of the Puuc relied almost completely upon water storage facilities fed by rainwater. Household cisterns, or *chultuns*–bell or beaker-shaped cavities lined with stucco to render them impermeable–were one solution. *Chultuns* were common during the Late-Terminal Classic period, although earlier settlement is too poorly known to definitively state when they were first used. *Chultuns* were excavated into the platforms of house compounds or into platforms especially made for the purpose [68, 69]. The upper surfaces of such platforms were stuccoed and slightly inclined so as to drain rain into the *chultuns*. The narrow *chultun* neck, about 50–70 cm in diameter and probably capped most of the time, prevented too much evaporation or the entry of debris. What is astounding is that 90–95% of the mouths of *chultuns* in areas that have been ground checked can also be seen in the lidar imagery (Fig 21). Others, having collapsed, are even more apparent.

**Fig 22:**
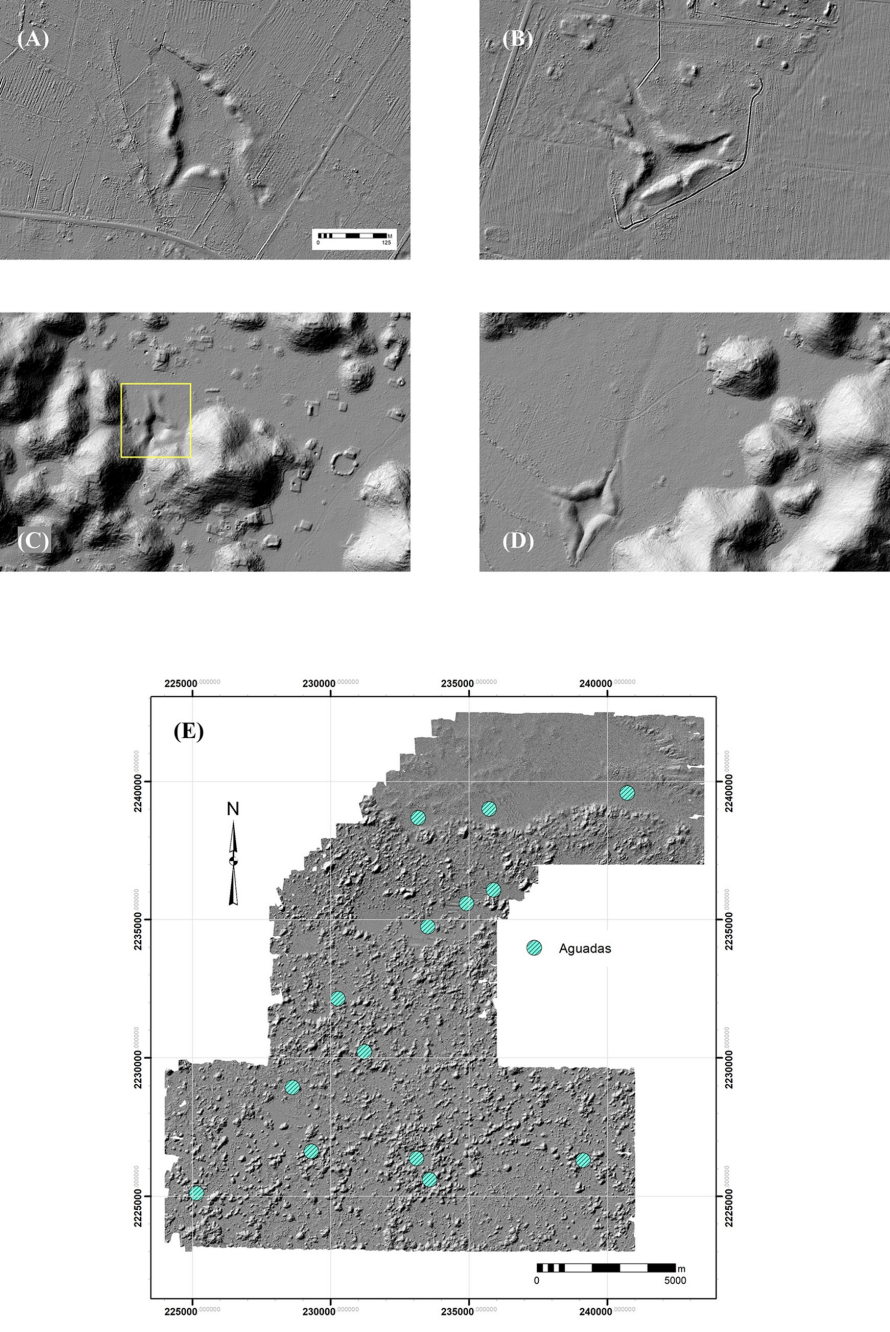
Lidar images of *aguadas*. (A) Aguada Xpotoit, Yaxhom. (B) Aguada Xloch, Muluchtzekel. (C) Kom *aguada*. (D) Huntichmul *aguada*. (E) *aguadas* identified in lidar imagery. Note feeder channels to *aguada* beds in examples A-D.

Another, perhaps older, technique of water storage was the construction or modification of communal *aguadas*, depressions modified for water storage by the construction of berms around their peripheries (Fig 22). We have identified fourteen of these in our regional sample and tentatively nine in the Alianza dataset. The dendritic forms of the larger examples result from feeder channels draining into the *aguada* beds, breaching the berms encircling them. Although difficult to detect on the ground, lidar reveals that such channels can be several hundred meters in length, greatly enlarging the drainage area of given *aguada*. Even the smaller examples usually have at least one such channel, although a few are simple depressions. The fact that the berms have almost no stone mixed in their fill indicate they were man-made, although they may have taken advantage of existing fractures. Some were lined with stone pavements while others have additional storage chambers excavated in their beds for the driest times of the year (*buk’te*s, first reported by Stephens [1]). Test excavations in one of the berms of the Aguada Xpotoit, within the site of Yaxhom, recovered ceramics mixed in the fill as far as our test pits reached, about 2 m below the surface, of which the great majority date to the Middle Formative. In contrast, Gallareta [38] reports only Late-Terminal Classic Cehpech sherds from a pit in the Labna *aguada*. Several pits in the bed of the Kom *aguada* also yielded only Cehpech sherds, suggesting *aguada* construction persisted throughout the prehispanic occupation of the Puuc.

*Aguadas* are confined to *planadas*, although in some cases these are quite small. Most of the major sites have *aguadas*, and their volumes generally track with the size of the settlement, Yaxhom, Muluchtzekel, and Huntichmul having the three largest. Some, however, are not directly connected with any site. Interestingly, there seems to have been some effort to prevent settlement near *aguada* beds: only 19 platforms were within 100 m of an *aguada* and most of these were in elevated *terreno intermedio*. As for access, 2434 (30.8%) platforms were within 1 km of an *aguada*, 5432 (64.2%) within 2 km. It is therefore interesting that just over a third of the platforms did not have ready access to an *aguada*, although the possibility of *aguadas* beyond the boundaries of our lidar survey must be taken into account.

GIS analysis of lidar DTMs permits approximation of current *aguada* capacities (Table 9), which can in turn be used to estimate how many people they could have sustained using daily consumption rates based upon ethnographic data [68, 70]. We developed a custom tool utilizing ArcGIS’s hydrology toolbox, especially its cut-fill tool, to estimate *aguada* capacities. (Briefly, rectangular shapefiles each large enough to entirely cover a given aguada are created to limit the extent of volume calculations. The cut-fill tool, in conjunction with the fill tool, calculates volume by detecting depressions, filling them, and subtracting the difference. The rest of the tool involves adapting it for cycling through multiple *aguadas*, converting raster outlines of *aguadas* to polygons for areal determinations, and then attaching statistics to these by means of zonal statistics.)

**Table 9:** Aguada metrics.

Name	Capacity (l)	Surface Area	Depth	Person-days water #1	Person-days water #2	Person-days water #3
Yaxhom	21,863,372	20,926	1.98	4,554,869	1,286,081	818,853
Muluchtzekel	11,151,727	12,087	2.10	2,323,277	655,984	417,668
Huntichmul	8,952,488	3,380	5.08	1,865,102	526,617	335,299
NN-Second planada E	8,923,870	4,671	4.22	1,859,140	524,934	334,227
Sabana Piletas	6,331,188	2,077	6.54	1,318,998	372,423	237,123
Kiuic north	3,814,771	1,457	5.67	794,744	224,398	142,875
Kom	3,307,523	1,371	5.48	689,067	194,560	123,877
Labna	1,696,620	1,267	2.49	353,462	99,801	63,544
Xkambul west	1,426,130	1,218	2.37	297,110	83,890	53,413
H-Wasil	972,487	1,623	2.71	202,180	57,205	36,422
Yaxhom Valley W	749,115	1,497	0.80	156,066	44,066	28,057
NN-Second planada S Center	697,374	897	1.35	145,286	41,022	26,119
NN-Second planada Center E	591,700	698	1.58	123,271	34,806	22,161
Xcalotpec SW	499,423	702	2.09	104,046	29,378	18,705
Totals	70,977,788			14,786,618	4,175,165	2,658,343

Consumption estimate 1: McAnany 1990, 4.8 l/person/day; Consumption estimate 2: Becquelin and Michelet 1994, average estimate, 17.0 l/person/day; Consumption estimate 3: Becquelin and Michelet: upper average consumption figure, 26.7 l/person/day.

Because of siltation and erosion, aguada capacities are probably underestimates, but are nevertheless informative. Comparative statistics indicate they are by no means equal, the Aguada Xpotoit (Yaxhom) far surpassing its neighbors at nearly 22,000 m^3^ while the smallest range closer to 500 m^3^. (By comparison, the volume of the Palace Reservoir of Tikal is about 31,000 m^3^ [19].) Overall, regional *aguada* capacity is estimated at about 71,000 m^3^ or 71 million liters. The Aguada Xpotoit, almost 300 m in length, accounts for about 31% of this total, and cumulatively the top four *aguadas* have 72% of total capacity.

Both McAnany [68] and Becquelin and Michelet [70] have used estimates of daily per capita water consumption and the number and capacity of *chultuns* to provide insights into population levels. McAnany used a figure of 4.8 l/day/person of any age, derived from a doubling of the world-wide estimate for minimum daily water consumption. In contrast, Becquelin and Michelet proposed an average minimum consumption of 17 l/day/person of any age and an upper estimate of 26.7 l/day/person of any age, based upon ethnographic observations. McAnany’s population estimates are therefore 3.5–5.5 times that of Becquelin and Michelet. Application of these estimates to our *aguada* capacities is provided in Table 9. Overall regional capacity would be 14.8 million person-days of water by McAnany’s estimate or from 2.7–4.2 million person-days using Becquelin and Michelet’s more conservative estimates, in either case a substantial amount. Supposing that the *aguadas* were the sole source of water for a 160-day dry season and neglecting evaporation (though clearly non-negligible), regionally a total of 92,400 people could have been supported using the 4.8/l/day rate or 16,600–26,100 using the 17–26.7 l/day figures.

Clearly, these are crude figures, and a more sophisticated model would track the rate of refilling of the *aguada* during the rainy season as well as evaporation rates, but they do provide a rough indication of the number of people who could have been sustained by *aguadas* alone. Rounding up the average *chultun* capacities analyzed by Becquelin and Michelet to 36 m^3^/*chultun*, regional *aguada* capacity would be the equivalent of around 1970 *chultuns*.

### Movement

**Fig 23:**
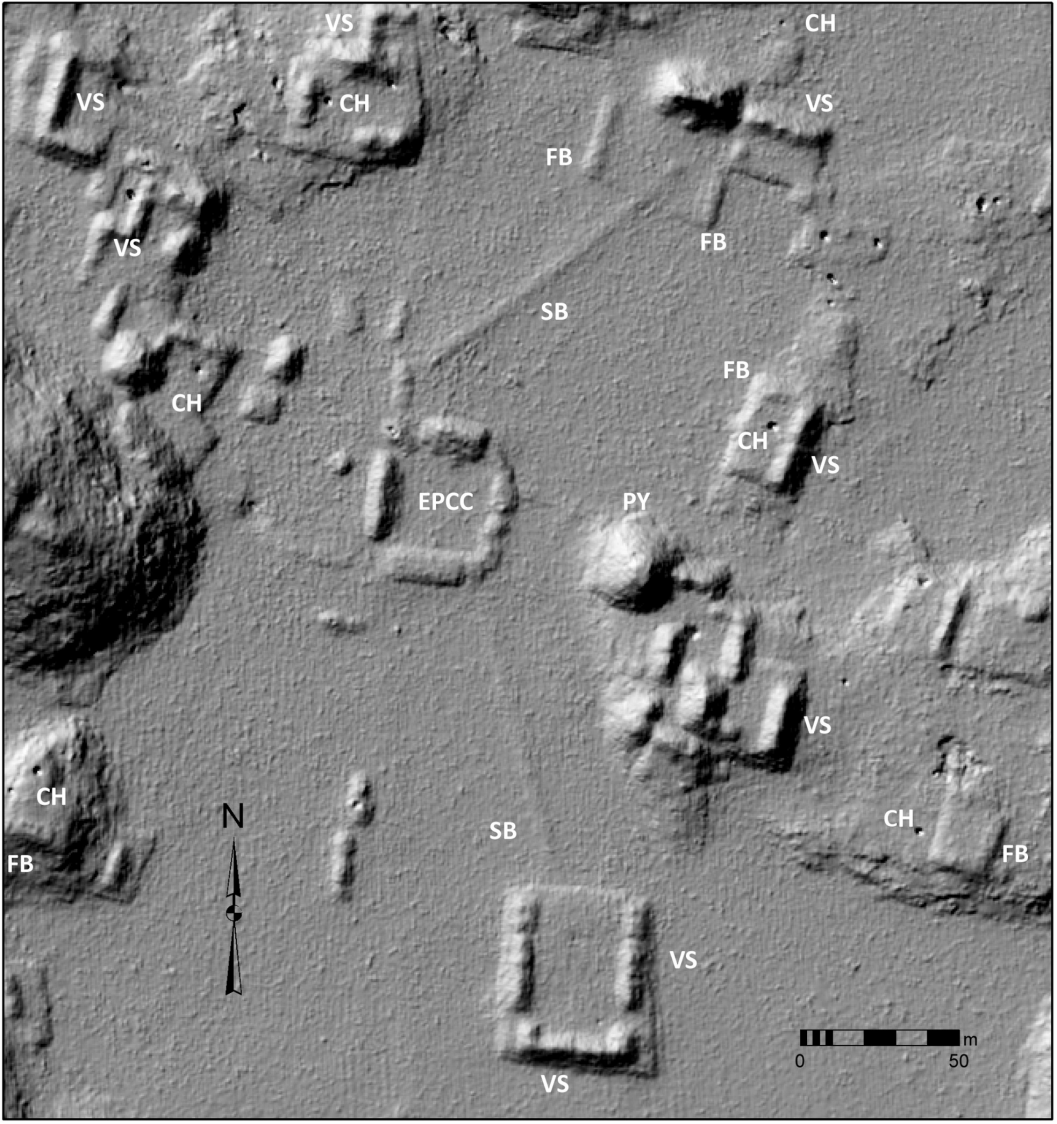
Sacbes (stone causeways) in lidar imagery of Caanil Cah. Note the connection of the earlier EPCC with later complexes with vaulted architecture. SB = sacbe (causeway), EPCC = Early Puuc Civic Complex, PY = pyramid, FB = frame brace, CH = chultun, VS = vaulted building. Not all instances are marked.

Landscapes are experienced dynamically, so an interesting question concerns the choices people made in moving through the various subregions of the Puuc. Stone causeways, or *sacbes*, are direct indicators of movement but generally Puuc *sacbes* are internal to sites, relatively short, and linear in bearing (the Uxmal-Nohpat-Kabah *sacbe* being the major intersite exception). Lidar proved quite capable of detecting linear features such as *sacbes*, despite the fact that in some cases they were almost invisible during field reconnaissance (Fig 23). As might be expected, the causeways in our sample are internal and relatively short given the terrain–mention has already been made of *sacbes* connected to EPCCs. Yaxhom is the exception proving the point, for although it was the terminus for a number of longer intersite causeways, most crossed relatively flat terrain. We found evidence of *sacbes* extending to the south, east, and north, in all cases leading to a civic group terminus (to the east, for instance, is the Middle Preclassic Acropolis Lakin). A further two causeways lead from Nohoch Cep, the southern terminus of the southern *sacbe*, to Nucuchtunich and a palace known as Cooperativa A. Although these causeways cross *terreno intermedio*, they are less than 1.5 km in length.

**Fig 24:**
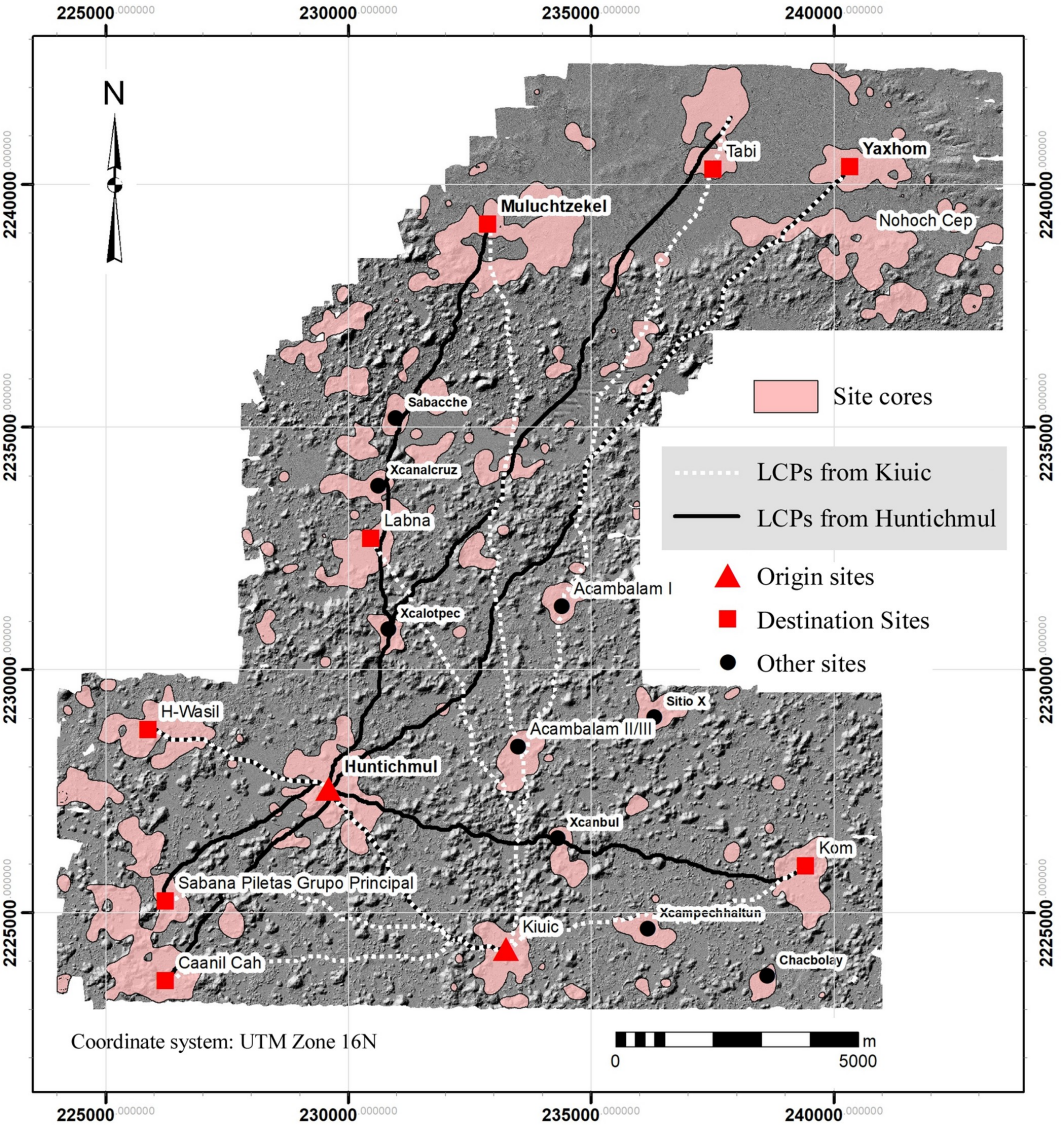
Least Cost Paths (LCPs) emanating from Kiuic and Huntichmul to eight other major destinations in the survey sample.

People must instead have generally traveled along now-lost paths. The number and relatively small size of the Puuc *cerros* presented an almost infinite number of ways to move across and over this landscape, in contrast to the relatively obvious choices across the northern plains, along the mountain valleys of southern Belize, or down river channels, where they exist. Because Puuc pathways are not obvious, one way to decide amongst the possibilities is to determine least-cost (or least-effort) paths (LCPs). A LCP is calculated by specifying a point of origin, one or more destinations, and a cost surface connecting them. In our case we calculated two networks, originating at Kiuic and Huntichmul respectively, both with destinations at Muluchtzekel, Tabi, Yaxhom, Labna, Kom, Caanil Cah, Sabana Piletas, and H-Wasil. Additionally, rules can be specified (such as to never cross bodies of water), but since there are few or no barriers in our study area, we use only a cost surface based upon slope. To better approximate walking energetics, we applied Tobler’s hiking algorithm to the DTM altitude raster and used that as the base for determining the cost of movement [71]. This curve reflects the fact that the energetically least costly movement is moving slightly downhill, beyond which costs again rise. We also resampled the original 0.5 m raster cells to 5.0 m cells to speed processing and even out small irregularities that a pedestrian could easily have avoided. Fig 24 shows the paths connecting the eight destinations to Kiuic, in dark red, and to Huntichmul, in black.

The problem then becomes to determine whether such paths were actually used. It might be expected that settlement would have grown up along intersite paths, so one test is whether platform density is elevated along them, as Hutson et al. [13] demonstrate along the Uci-Kansacab causeway. To test this, we calculated the number of platforms within 100 m and 200 m buffers along the LCPs to Kiuic, to Huntichmul, and then with respect to the combined LCPs (Table 10). Overall density within the buffers is 47 platforms/km^2^, about 41% over the average study sample density. If LCPs attracted settlement, we would also expect settlement density to decline with increased distance to the path. Although this is upheld in both the Huntichmul and Kiuic datasets, because they intersect and overlap somewhat, the best test is the combined dataset. In this dataset, settlement (number of platforms) from 100–200 m away from the LCP was 76% that of settlement within the 100 m buffer. These figures therefore support the hypothesis but are not overwhelmingly strong. In fact, when only the sectors of the LCPs that lie outside the site cores are considered, there is no appreciable difference between the two buffer zones, platform density within the buffers being about that outside them. Thus, whatever attraction proximity to LCPs had, it was mostly within the site cores.

**Table 10:** Settlement along least-cost paths.

LCP&Buffers	Area (km^2^)	% Total Area	Platforms	% Total Platforms	Platform Density	Annulars	% Total Annulars	Annular Density
**Kiuic**								
100 m buffer	15.12	6.4%	767	9.7%	50.72	80	6.5%	5.29
200 m buffer	14.71	6.2%	706	8.9%	47.99	94	7.6%	6.39
combined	29.83	12.6%	1473	18.6%	49.38	174	14.1%	5.83
**Huntichmul**								
100 m buffer	15.01	6.3%	893	11.3%	59.50	101	8.20%	6.73
200 m buffer	14.51	6.1%	731	9.3%	50.38	103	8.36%	7.10
combined	29.52	12.4%	1624	20.6%	55.01	204	16.56%	6.91
**Combined**								
100 m buffer	25.00	10.5%	1264	16.0%	50.57	148	12.01%	5.92
200 m buffer	22.35	9.4%	960	12.2%	42.95	144	11.69%	6.44
combined	47.35	20.0%	2224	28.2%	46.97	292	23.70%	6.17
**Total sample**	**237.23**		**7902**		**33.30**	**1232**		**5.19**

A better test is whether LCPs pass through communities not defined as destinations. Three destination sites to the southwest (H-Wasil, Sabana Piletas, Caanil Cah) are too close to Huntichmul to have intermediate sites of any size along the LCPs, though the path from Kiuic to H-Wasil does pass through Huntichmul. The path connecting the southeast destination site of Kom to Huntichmul passes through the intermediate site of Xcanbul, while to the south the path from Kiuic to Kom passes through Xcampechhaltun. To the north, the site of Acambalam II/III is interesting because the paths from Kiuic to Muluchtzekel and to Tabi branch exactly at its center. Another branch to Labna along this LCP diverges somewhat to the south but may originally also have left from Acambalam II/III. The LCP from Kiuic to Tabi also goes through Acambalam I and an unnamed population cluster at the east end of the Sabacche *planada*. The LCP to Muluchtzekel goes through several small population clusters to the east and northeast of Labna. With regard to the Huntichmul LCPs, another crossroads is the small site of Xcalotpec, where the LCP to Muluchtzekel diverges from the path to Tabi. The western branch, to Muluchtzekel, also passes through Labna and another elongated sprawl of settlement extending to Sabacche and the western edge of the Sabacche *planada*.

**Fig 25:**
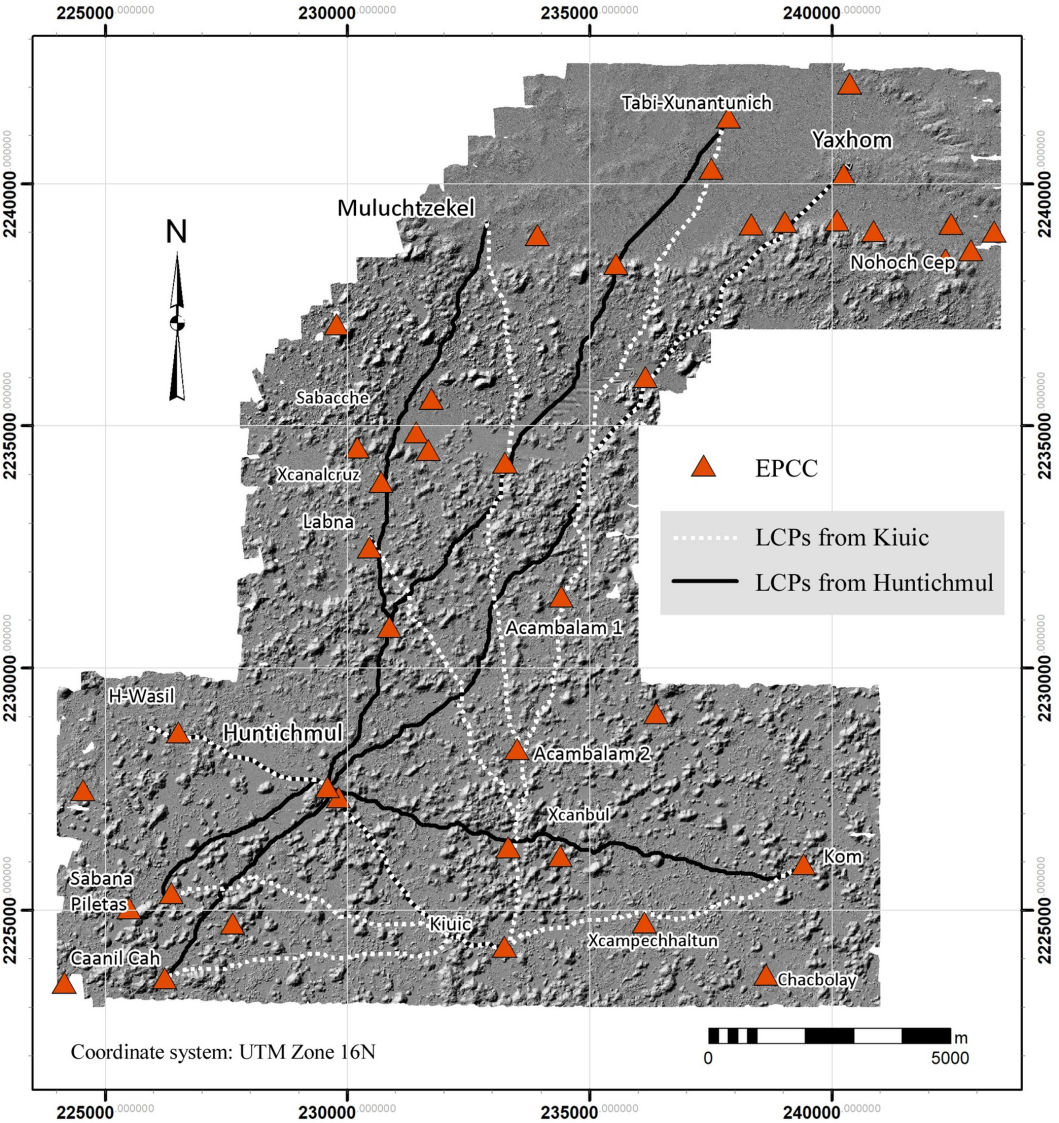
Spatial relation between EPCCs and LCPs.

Because of the perturbing edge effects of our irregularly shaped lidar sample, and because of its limited extent, we also calculated LCPs to neighboring regional sites using the 30-m-resolution DEM ASTER dataset (https://asterweb.jpl.nasa.gov/gdem.asp). These are obviously much coarser than the lidar surface and are effectively DSMs, but calculated LCPs do generally follow the finer lidar LCPs. Of interest are paths to Chacmultun, a substantial center to the east. The one leading from there to Huntichmul also passes through Acambalam II/III, reinforcing its importance as a crossroad, and then continues to the northeast to pass through a substantial unnamed cluster labeled “Sitio X”. The path from Chacmultun to Kiuic passes directly through Kom and then Xcampechhaltun. A related metric is the proximity of EPCCs to LCPs. Several of the EPCCs are beyond the destinations, and so are irrelevant to this test (such as those around Yaxhom and to the west of Sabana Piletas), but of the 20 or so that do lie between destination and origin points, at least two-thirds are very close to LCPs (Fig 25).

Archaeological evidence can increasingly be added to the search for past travel routes. For instance, two large unshaped upright monoliths were erected at places where distinct LCPs entered Huntichmul, while at Kiuic the LCP from Huntichmul was marked by a ramp, perhaps functioning as a check point. Gallareta and May have also noted that isolated *chultun* platforms were found along the LCP connecting Kom with Xkambul and Huntichmul, perhaps places where travelers could have refreshed themselves or filled their water gourds for the journey. They have also identified possible vantage/surveillance points along the route from Labna to Huntichmul, as has Ringle along the route north from Kiuic. We anticipate more intensive inspection of the lidar and additional fieldwork will strengthen the case for these LCPs.

## Discussion

### Puuc demography

After an earlier peak of interest in the 1980s and early 1990s, following the results from an initial wave of settlement studies [72], Maya demography has again surged to the forefront of Maya studies in the wake of several large lidar campaigns (e.g., [19, 73]). One reason for a falloff of interest in intervening years was the fact that most settlement studies had been site focused and hence concentrated in areas of peak population density. There were notable exceptions, such as the Copan valley and the Peten Lakes area, but comprehensive statements concerning comparative demographics were all but impossible without a better sense of overall population levels, especially with regard to the organization of the rural population.

Lidar imagery has gone a long way in removing that limitation, but several of the problems involved in archaeological demography have yet to be resolved, as noted above with regard to estimates based upon agricultural production and water storage capacity, already articulated by Culbert and Rice [72]. A direct calculation of ancient population levels on the basis of structural remains usually depends on several factors, as outlined by Tourtellot et al. [48] for Sayil. First is a count of the number of structures presumed to be houses or households. An alternative strategy might employ the number of habitable rooms or the floor area of dwellings, where such data can be gathered, implying the exclusion of buildings or rooms that served other purposes, such as field huts, kitchens, storage areas, etc. Second, there is the question of the size of the social unit associated with structures, which might be a nuclear family, an extended family, or a household. These are usually derived from ethnohistorical or ethnographic accounts, which vary greatly, from 4.5–13.5 in cases from the colonial Maya world (see [74] for a list). The third necessary parameter is an estimate of how many structures were being occupied contemporaneously. This is perhaps the most difficult to determine archaeologically, since even if dwellings are dated by pottery, it cannot be assumed they were inhabited for the full length of a given ceramic phase. Furthermore, buildings were not infrequently enlarged during their life history, a process which usually can be established only by excavation. In lieu of that, buildings are usually tallied in their final form.

This has led to estimates which are inflated, in our view. For instance, Tourtellot et al. [48] estimated the population of the nearby Puuc site of Sayil at 10,858, in general agreement with McAnany’s [68] estimate of water capacity, which, as noted, has been criticized for using very low estimates of daily personal consumption. Tourtellot et al.’s estimate can also be criticized on a number of levels. They assume that settlement density in the non-mapped portions of the site was equivalent to that of the site center, most probably resulting in an overestimate. Second, about 34% of the estimated population is argued to have resided on gravel mounds with no frame brace foundations (*chiches*). *Chich* mounds have never been conclusively demonstrated to be domestic features, and it is likely that many or most of the bare platforms were just that, loci for work activities. Since frame brace construction depends on gathering a modest number of small boulders that are everywhere available, it is difficult to believe that a substantial fraction of the population were too poor to make this most minimal investment in house construction.

Third, Tourtellot et al. elected to utilize room counts, assuming that each was occupied by something close to a small nuclear family (4 individuals/ room), whether perishable or of masonry. Using the average number of rooms per building type, perishable buildings were argued to have 6.6 residents on average and masonry buildings 13.52, because of their greater number of rooms. Our excavations of the Escalera al Cielo complex [43, 67], which was abandoned rapidly, demonstrates that rooms often were given over to storage and that small structures were probably outbuildings.

It seems more likely that each building of two or three rooms housed a single nuclear family, as perhaps did some of the larger one-room buildings, with platforms generally representing a single extended household (in fairness, an option also considered by Tourtellot et al.). Buildings with more rooms may have housed multiple nuclear (or extended) families, but they are relatively few in number. A calculation on this basis reduces the Sayil estimate to around 3000 people. The Sayil project also registered a total of 296 basal platforms. If we assume that each supported a single household, assumed to be equivalent to what is referred to as an extended family in the anthropological literature, and then employ a figure of 10 people/household, about midway in the range of values in the ethnohistorical literature, we arrive at much the same figure. Finally, Tourtellot et al. argue that Sayil was a single component site, so they made no adjustment for contemporary occupancy. This is almost certainly unwarranted, as they note, but it is also almost impossible to provide an accurate alternative. Even so, it seems that a population estimate closer to one-half (or less) of their total is more likely.

Another problem is that most lidar projects are focused around one or more known centers of significant size, whose structure densities may exceed by a significant amount random samples of settlement. This seems to be the case with the Pacunam datasets [19] (Canuto et al. 2018: Tables 1 and 4). The Tikal and Naachtun datasets, covering 146.9 and 135.3 km^2^ respectively, exceed 80 structures/km^2^. Five of the remaining survey blocks are of roughly comparable size, between 90–165 km^2^, and are also focused around known sites of substantial size, resulting in structure densities of around 30-45/km^2^. In contrast, three unnamed datasets to the west, taken as samples of unknown territory, cover a total 502.2 km^2^ with an average structure density of only 2.56/km^2^. The remaining two datasets, Corona and Holmul, are the two largest, covering 432 and 309 km^2^ respectively, but significantly their densities are only 8 and 23/km^2^. In short, the seven datasets measuring less than 165 km^2^ in area seem to be dominated by urban cores and nearby secondary centers, skewing the average higher, while the environmental samples and the two largest blocks show markedly lower densities. A further concern is that the Pacunam density estimates are directly extrapolated to the central Maya lowlands as a whole, a region of 95,000 km^2^, with little regard to variations of terrain and vegetation, although it encompasses significant expanses of seasonal swamps (*bajos*), the Maya mountains and other upland areas, and other regions which may have been less propitious for farming and urban development.

Our own sample suffers from a similar bias, since we were specifically interested in the interactions between several known sites distributed throughout the sample region. Some indication that it is not representative is supplied by the Alianza dataset, which extends only 7–8 km south of the NCALM dataset but displays a clear diminution of settlement. Sites are generally less frequent, less dense, and smaller in this dataset. A preliminary tally registers only 1327 basal structures; density is 11.4/km^2^ versus 33.3/km^2^ for the NCALM region. If the NCALM structure density isopleths are applied to this region, the two lowest-density sectors occupy 85.5% and 11.4% respectively of the total area of the new region, or 96.9% in total (vs. 83.5% for the NCALM dataset). Whether this southward decline in settlement continues awaits further survey, but it is in accordance with the fewer number of known sites in that direction.

For purposes of comparison with other areas, and with a recognition of our methodological limitations, if the total of 7902 basal structures identified regionally is adjusted by 125.3%, based upon the Muluchtzekel statistics of identified to non-visible basal structures, we arrive at a total 9900 basal structures in the regional sample of 237.2 km^2^, or 41.73/km^2^. At Muluchtzekel, 348 of the 525 platforms supported buildings, which, projected regionally, would result in an estimate of 6550 platforms that were potentially residential (27.6/km^2^). If we extrapolate from the 1021 buildings identified in the Muluchtzekel survey, we arrive at a total of around 19,200 buildings (81.0/km^2^) in the NCALM sample.

Table 11 presents a population estimate based upon a projection of how many buildings of a given room size would be present in the 19,200 buildings estimated to be in our regional NCALM sample. In this we make the assumption that buildings of a given size were occupied by an integral number of nuclear families (e.g., one nuclear family for a two- or three-room building). We also assume that only 62.3% of the single-room buildings housed a nuclear family. This is based upon the assumption that a minimum floor space of 8 m^2^ was necessary for a single-family house; the adjustment represents the ratio at Sayil for single-room buildings of this size versus all single-room buildings. (Thus, 77.5% of all buildings are projected to have been residences, for a density of 62.7/km^2^.) Finally, to arrive at people, we assume a nuclear family size of 5 and a 90% co-occupation ratio, mostly for compatibility with other estimates. The result is about 72,500 people. Some buildings of the final class were probably civic, rather than residential, structures, so rounding the estimate to 70,000 would yield about 295 people/km^2^.

**Table 11:** Population projected on the basis of room statistics.

No. of Rooms in Building	% of Buildings at Muluchtzekel	Projected Buildings in Region	Nuclear Families per Building	Projected Nuclear Families	Adjusted Population**
1	59.69%	11,460	1*	7,140	32,129
2	26.27%	5,043	1	5,043	22,696
3	9.88%	1,898	1	1,898	8,539
4	1.69%	325	2	649	2,922
5	0.78%	150	2	300	1,348
6	0.65%	125	3	374	1,685
7	0.39%	75	3	225	1,011
8	0.00%	0	3	0	0
9	0.13%	25	3	75	337
10+	0.52%	100	4	399	1,797
**Totals**		**19,200**		**16,103**	**72,464**

* A factor of .623 (62.3%) is applied to the projected 11,460 single-room buildings, derived from the frequency of single-room buildings 8 m^2^ or more at Sayil versus the frequency of all single-room buildings. The result is a total of 7,140 possible single-room residences and 4,320 service buildings.

**An average nuclear family is estimated to have five individuals. A co-occupancy value of .9 (90%) has also been applied to make value comparable to those derived elsewhere.

Another approach is to assume that a platform was occupied by a single household, thus the maximum population would equal the number of residential platforms multiplied by an average value for household size. Using the estimate of 6550 possible residential structures, an estimate of 10 people per household, and a co-occupation ratio of 90%, a total population of around 59,000 is indicated, about 20% less than the previous calculation. Obviously, the parameters used in both calculations can be disputed: some have used values of 4 for a nuclear family, others a value as high as 6. Similarly, household size estimates run as high as 13.5. The co-occupation ratio is also little more than an educated guess but does not differ greatly from other recent demographic estimates. A reasonable compromise range would be ±20%, or 56,000–84,000 people within our sample boundaries.

Other caveats warrant consideration. First, Muluchtzekel, given its urban character, is probably not representative in terms of the number and ratios of building types elsewhere; a better estimate will take into account other areas that have been ground verified. In other words, community size may ultimately need to be considered, since different extrapolation factors may be necessary for sites of different ranks. Second is the problem of buried structures, especially in the planadas where soils are deeper. Determining whether this was the case will require extensive (and costly) excavation programs. However, we note that the *terreno intermedio* does not differ greatly in the types of structures present, i.e., we did not encounter large numbers of modest structures of the type that might be buried in the *planadas*. Furthermore, some rather modest structures do appear in the *planadas*. Although few in number, they do indicate soil deposition was not excessive. Undoubtedly some structures are buried, but we argue on these grounds that the number is low. Completely perishable buildings are almost impossible to detect without a large investment in advanced methodologies, but we believe these two were a minor component of settlement, if indeed they existed.

This estimate is near the maximum calculated carrying capacity discussed above, indicating either that our direct estimate of population is too high, our estimate of carrying capacity is too low, or a combination of the two. Alternatively, the area may have been supplied from less densely settled regions nearby. With regard to water, *aguada* and *chultun* capacity appears to comfortably accommodate a population of around 70,000. This suggests the area was well-buffered again droughts, at least for household needs; watering crops is another story.

Projections to the Puuc region as a whole are equally problematic. If we take the line N2207000 as an arbitrary southern limit to the Puuc, it covers approximately 5275 km^2^, 3350 km^2^ of which belongs to the Bolonchen District, 1800 km^2^ to the Valle de Sta. Elena and about 125 km^2^ to the Sierrita de Ticul. In our regional sample, there is a marked difference in platform density between the VSE and DBC sectors, 40.7 vs. 25.8 platforms/km^2^. However, the former is dominated by the Valle de Yaxhom basin and its neighboring *planadas*, which are largely vacant, and we at present have no way of determining how typical this is of the remainder of the Valle de Sta. Elena. Similarly, our sample of the Bolonchen Hills may also be skewed. On the one hand, it lies in a hotspot of known sites (Fig 2), and, on the other, settlement apparently falls off to the south (Figs 8 and 9).

With regards to the lowlands as a whole, Ceibal provides a useful comparison of a central Peten site with similar settlement units to ours [75]. There 1041.7 platforms and 15,380.5 residential buildings are projected within the study area of 470 km^2^, resulting in densities of 2.21 platforms and 32.7 residential buildings per square kilometer. Our overall platform density is 41.7/km^2^, reduced to 27.6/km^2^ if only residential platforms are considered. Our building density is 81.0/km^2^ unadjusted or 62.7 residences/km^2^. Canuto et al. [19] cite an average structure density of 28.67 structures/km^2^ for the Pacunam Central Peten sample and estimate population density as roughly 80–120 persons/km^2^, but uncertainty over the basic units in these surveys makes comparisons difficult. However, this is eventually resolved, and whatever the error of our parameter estimates, it seems clear that our sample of the Puuc enjoyed a significantly higher population density than their Peten neighbors. With more refined projection factors, greater stratification of projection classes, and additional lidar sampling of the Puuc, even greater precision in population estimates can be expected.

## Conclusions

Returning to the questions raised at the beginning of this article, Lidar imagery reveals a landscape that was densely but unevenly occupied. Perhaps the most notable aspect of regional settlement distribution is the relatively sparse use of the hinterlands, including some areas of considerable size. Some are to be expected, such as the cluster of hills north of Kiuic and south of Acambalam II/III, but others are less easily explained, such as the large expanse west of Kom, or that south of Huntichmul and west of Kiuic. Most intriguing is the mid-central *planada* where Sabacche is located. Although the *planadas* of the Valle de Yaxhom are the most densely settled areas of our study region, the eastern half of the *planada* is very lightly settled and has relatively few civic structures, despite its apparent fertility, judging from modern cultivation.

At the other extreme are the many communities dotting the landscape. Thirty-one of the 106 site cores (Sectors 3–5) have 38 or more basal structures and a minimum size of 27 ha (if Yaxhom is treated as a single extended conurbation, the number of site cores declines to 28). This would translate to an average realm covering only 7.7 km^2^, which, if they were packed hexagons, would have centers just under 3 km apart. This agrees well with the actual distances between cores and their nearest neighbor, which do not exceed 4 km, even if restricted only to the largest 31. Distances are often half that, reflecting the fact that 16 core areas have a hundred or more platforms and so were many times larger than their neighbors. Below the 31 largest site cores, there is a significant break both in the size of and the number of platforms within core boundaries.

A model of discrete civic domains, with relatively concentric settlement gradients, can only be sustained in some cases. In other cases, the amorphous distribution of Sector 2 settlement envelopes multiple civic complexes, hinting at a more complex form of urban organization. The largest such distribution is Yaxhom, discussed earlier, but the areas around Muluchtzekel, Labna, and Sabana Piletas are similar. The latter is particularly unusual in having not only several lobes of settlement but also at least two civic complexes. Settlement extends continuously to encompass Caanil Cah as well, which has its own well-developed civic center. The settlement extending north from Labna includes the smaller centers of Xcanalcruz and Chuncatzim, and then extends to the sites around Sabacche. This suggests that settlement fall-off alone cannot be used to define sites.

The significant differences in the distribution of settlement most probably reflect constraints on settlement choice. These may have been the result of agreements between adjacent centers concerning the cultivation of *planadas* and other resources, perhaps similar to the indigenous agreements described in sixteenth-century native documents such as the *Land Treat of Mani* [76] and the *Codice de Calkini* [77]. Limited settlement around the *planadas* and in the hinterlands implies that population growth had not yet reached a demographic crisis when the region was abandoned during the tenth century, also supported by the very limited use of agricultural terraces. The cause for this abandonment remains contested, but internecine warfare does not seem to be the proximate cause, given the close packing of communities and the absence of defensive works. Settlement does drop off significantly to the south, however, perhaps because it was more contested territory.

The low level of settlement on *cerros* also indirectly suggests that considerable areas remained forested. It is true that about half of the annular structures, presumably stoked with wood from nearby forests, do lie within the two lowest platform density sectors (Table 8), yet the density of annular structures is quite low, given that these two sectors comprise 83.5% of the study area. In fact, annular density within the lowest platform density sector, Sector 1, comprising 63.3% of the study area, is less than a third of Sector 2. Although the use of a fuel-efficient lime pit-kiln technology suggests some concern with forestry management [64, 66], nearly half the annular structures were within communities, suggesting fuel was available nearby.

As to the question whether the Puuc had a distinctive civic arrangement, the frequency of EPCCs throughout our study region suggest that regional complexity grew out of a series of small centers that were remarkably similar. Only one EPCC does not fall within a site core as defined by settlement density isopleths, and 37 (88%) are located within the 31 largest site cores, although four of these cores lack an EPCC. Six site cores had more than one EPCC, reflecting later settlement consolidation. EPCCs probably reflect the initial importance of councils in early Puuc governance, a trend we argue continued throughout the Classic period [78]. With regard to later civic complexes, while it is clear that civic organization took a variety of forms during the Terminal Classic, some unique to particular sites, it is also clear that palaces often assumed a relatively limited set of forms, but that is a topic of future discussion.

The influx of new data stemming from the lidar revolution has clearly enhanced our ability to address fundamental questions concerning Puuc settlement and urbanism, sometimes confirming past arguments but in others presenting entirely new vistas [79]. With regard to comparisons elsewhere, although we generally agree with Stanton et al. [15] that the volume and area of platforms “have the potential to be more easily comparable to other datasets based on structure density,” thus avoiding the problem “that one large pyramid ‘counts’ the same as one small ancillary building in a domestic group,” it is clear that multiple, multi-factor lidar classifications will ultimately be necessary, since a small house may indeed outweigh a large non-domestic structure if demography is the question. Our review of the imagery continues and will eventually employ a structure typology developed by Gallareta and May, allowing better estimates of the number of buildings and hence an improved idea of Classic-period demography. We also expect a more precise assessment of the accuracy of feature identification from the lidar imagery alone, allowing better extrapolation into unsurveyed areas. Finally, we are in the process of applying additional GIS techniques, particularly to help us model visual communication within and between sites.
